# Optimising Health Literacy and Access (Ophelia) in Florence, Italy: a cluster analysis to guide cardiovascular prevention among perimenopausal and postmenopausal women in a vulnerable urban setting

**DOI:** 10.1136/bmjopen-2025-115177

**Published:** 2026-07-16

**Authors:** Claudia Cosma, Guglielmo Bonaccorsi, Claudia Biagi, Chiara Milani, Giulia Naldini, Diletta Buresta, Gabriele Cerini, Alice Graziani, Marco Del Riccio, Patrizio Zanobini, Michele Volpi, Veronica Gallinoro, Lorenzo Baggiani, Marco Nerattini, Chiara Lorini

**Affiliations:** 1 Department of Health Science, University of Florence, Florence, Tuscany, Italy; 2 Health Literacy Laboratory, Department of Health Science, University of Florence, Florence, Tuscany, Italy; 3 Department of District Healthcare Network, Azienda USL Toscana centro, Florence, Italy; 4 Medical Specialization School of Hygiene and Preventive Medicine, University of Florence, Florence, Italy; 5 Health Society of Florence, Florence, Italy

**Keywords:** PUBLIC HEALTH, Aging, Behavior, Cardiovascular Disease, Chronic Disease

## Abstract

**Abstract:**

**Background:**

Cardiovascular risk increases markedly in perimenopausal and postmenopausal women and social disadvantage can intensify barriers to prevention. In these settings, limited health literacy may further restrict women’s ability to understand cardiovascular risk, access appropriate services and translate advice into daily behaviours—potentially widening inequalities.

**Objectives:**

This study investigated health literacy profiles among perimenopausal and postmenopausal women living in a socioeconomically vulnerable urban area of Florence, Italy, and examined how these profiles may guide locally tailored cardiovascular prevention strategies using the Ophelia approach.

**Design:**

Cross-sectional study including hierarchical cluster analysis of Health Literacy Questionnaire (HLQ) scores.

**Setting:**

Primary and community health services in a socioeconomically vulnerable neighbourhood of Florence, Italy.

**Participants:**

Women aged 45–70 years attending the House of the Community ‘Le Piagge’ between October 2024 and January 2025. Of 188 recruited, 156 provided complete HLQ data and were included in the analysis.

**Primary and secondary outcomes:**

Primary outcome: multidimensional health literacy profiles (nine HLQ scales). Secondary outcomes included Nutrition Literacy Instrument, adherence to the Mediterranean diet (MEDI-LITE) and Sense of Coherence.

**Results:**

Four distinct health literacy profiles emerged (p<0.001). One profile (16.7%) showed broad strengths across HLQ domains, another (48.7%) combined several strengths with some more selective challenges, a smaller profile (8.3%) showed widespread challenges across domains, and a fourth profile (26.3%) showed mixed strengths and challenges together with more limited social support. Profiles characterised by more marked health literacy challenges were associated with lower education, greater financial difficulty, poorer self-rated health, lower nutrition literacy and weaker adherence to the Mediterranean diet. Among the Sense of Coherence dimensions, meaningfulness was higher in clusters with stronger health literacy profiles (p<0.001).

**Conclusions:**

This study identified distinct multidimensional health literacy profiles among perimenopausal and postmenopausal women in a socioeconomically disadvantaged urban setting. These profiles provide locally relevant evidence to inform the subsequent co-design of fit-for-purpose cardiovascular prevention actions within the Ophelia framework.

**STRENGTHS AND LIMITATIONS OF THIS STUDY**

- Use of a validated multidimensional health literacy tool (Health Literacy Questionnaire), enabling detailed profiling across nine domains.
- Integration of the Ophelia approach to link quantitative results with locally relevant, equity-oriented interpretation.
- Inclusion of complementary constructs related to nutrition, lifestyle and psychosocial resources, allowing richer characterisation of the study population.
- Recruitment in a single socioeconomically vulnerable urban setting may limit generalisability.
- The cross-sectional, descriptive design limits temporal interpretation of the observed associations.

## Introduction

 Cardiovascular diseases (CVDs) are the leading cause of death worldwide, causing 17.9 million deaths per year, accounting for 32% of all deaths globally. Over 75% of deaths related to CVDs occur in low- and middle-income countries. However, even in high-income countries, there are significant social and geographical disparities in access to prevention and management of risk factors. Although the individual risk of developing CVD has decreased over time in the West, the total number of cases and deaths remains high due to an ageing population and persistent exposure to modifiable risk factors such as high blood pressure, poor diet, physical inactivity and environmental pollution.^1 2^ In Europe, CVD has a particularly significant impact on perimenopausal and postmenopausal women.^3^

The menopausal transition is a critical time for women’s cardiometabolic health. The sharp decline in oestrogen during this stage causes women to lose the vascular protection they had during their fertile years, which raises their risk of CVDs.^4^ In fact, menopause is associated with an increased prevalence of obesity, metabolic syndrome and CVDs. In such a peculiar period of women’s life, lifestyle factors—mainly nutrition and physical activity—can reduce the risk of developing all the diseases. In particular, the Mediterranean Diet (has been highlighted for its potential in providing greater protection against CVDs, either for primary or secondary prevention.^5^ As stated by the European Menopause and Andropause Society, evidence from observational studies and randomised controlled trials suggests that the Mediterranean Diet has positive effects on short-term and long-term menopausal health, including CVD.^6^ Nonetheless, in Europe, adherence to the Mediterranean diet is gradually decreasing, particularly among socially disadvantaged populations.^7^

Among the determinants of cardiovascular health, an important role is assigned to health literacy (HL), defined as ‘the ability of individuals to access, understand, evaluate and apply health-related information to make informed decisions in their daily lives’.^8^ Insufficient HL is associated with low consumption of fruit and vegetables, low level of physical activity, low adherence to treatment and prevention services, insufficient control of risk factors and increased premature mortality.^9 10^ Moreover, nutrition literacy, which refers to the capacity to understand and apply nutritional information to make healthy eating choices, is especially pertinent in the context of the prevention of CVDs: it encompasses specific food-related knowledge and is an extension of HL’s application skills.^11^

In response to the challenges of CVDs, the European Commission’s JACARDI (Joint Action on Cardiovascular Diseases and Diabetes) initiative aims to strengthen coordinated actions in 18 member countries directed at reducing the overall burden of chronic noncommunicable diseases and health inequalities. Through 142 pilot studies, JACARDI encompasses the whole ‘patient’ journey, including health promotion, primary and secondary prevention, improved service pathways and (self-)management.^12 13^

In line with the most recent international literature, JACARDI promotes strategies that actively involve the community.^13 14^ Promoting participation and strengthening health systems has been the response of the Pan American Health Organization as well, including in response to gender inequalities in cardiovascular health.^15^ In fact, one of the most frequent limitations of public health and health promotion interventions lies in their excessive standardisation, often based on the so-called ‘average user’ and not sufficiently adapted to the real and diverse needs of communities. Traditional top-down public health interventions often fail due to complex and context-specific factors. This leads to a loss of effectiveness and a worsening of inequalities, with the risk of leaving behind the most vulnerable groups.^16^ Complex health needs, resulting from the influence of factors varying between individuals and specific settings, require tailored, localised solutions. Co-creation—collaborative development involving academics, end-users and stakeholders—empowers participants, increases adherence and generally enhances effectiveness by also involving those who are generally left behind.^17^ The Ophelia (Optimising Health Literacy and Access) approach, developed in Australia and applied in numerous international contexts, fits into this theoretical and operational framework. It has been recognised by the WHO as useful for the development of HL in the perspective of preventing and controlling noncommunicable diseases.^15^ It is a participatory framework that combines needs analysis with the direct involvement of the target population (co-design) in order to develop tailor-made interventions. The Ophelia process uses three phases and eight steps^18 19^:

1. identify strengths, needs and action ideas (data collection and cluster analysis to identify HL profiles)—including three steps;
2. select, plan and test HL actions (co-design with target groups to identify barriers, resources and shared strategies)—including three steps;
3. implement and evaluate and improve HL actions—including two steps.

In order to effectively address the prevention needs and reduce health inequalities, the JACARDI pilot project ‘Individual and organisational health literacy as key factors to prevent cardiovascular diseases among general population with a focus on disadvantaged people in primary healthcare settings: a pilot study in Florence using the Ophelia approach—Ophelia in Florence*’* has adopted the Ophelia model, with a focus on women aged 45–70 engaged with the *Casa della Comunità* (‘House of the Community’) ‘Le Piagge’, located in a socioeconomically disadvantaged urban area of Florence. Recruitment was conducted among users of the *Casa della Comunità* ‘Le Piagge’; residence in the neighbourhood was not an inclusion criterion, and some participants lived elsewhere. Thus, the study is contextualised within the service catchment of Le Piagge rather than strictly its resident population. The project aims at giving women knowledge and skills to increase their level of HL, nutrition literacy, as well as their adherence to Mediterranean Diet, combining individual and organisational actions.

In this context, this paper aims at presenting the results of the first two steps of the Ophelia process, which are those devoted to the quantitative evaluation of HL, knowledge and behavioural needs of the target group.

## Methods

### Setting and participants

This study was conducted in *Le Piagge*, a socioeconomically disadvantaged urban area of Florence, characterised by a higher deprivation index than the municipal average, poorer health outcomes, particularly in relation to CVDs, and a diverse population, with approximately 15% of residents being foreign-born. Specifically, the population of *Le Piagge* is characterised by a markedly higher proportion of highly deprived residents (47% vs 25% in the Municipality of Florence), as well as higher hospitalisation rates (approximately 142 per 1000 inhabitants vs 121 in the Municipality of Florence) and higher mortality rates among women (947 per 100 000 inhabitants vs 680 in the Municipality of Florence).^20^ The community benefits from a network of services anchored in the *Casa della Comunità*, a primary healthcare facility of the Local Healthcare Unit (LHU) which provides general practice, nursing, pharmaceutical, maternal and child health, dentistry, rehabilitation, vaccination, and health promotion services.^21^ The *Casa della Comunità* Le Piagge is attended mainly by people living in the Le Piagge area, but also by residents from other parts of District 5—that is the district in which Le Piagge is located—and from other Districts of Florence.

### Project set-up and data collection

Phase 1 of the Ophelia process includes project set-up, local data collection, and stakeholder/community engagement.^19 22^ The present paper reports the cross-sectional quantitative profiling component of this broader ongoing Ophelia project, corresponding to the initial data collection stage. The overall project started on 1 December 2023 and is ongoing. Participant recruitment and questionnaire data collection for the present study were conducted between October 2024 and January 2025. The qualitative and co-design phases of the Ophelia process have already been conducted and will be described in a subsequent paper.

#### Project set-up (December 2023–September 2024)

Following the Ophelia manual,^19^ focus (the issue we want to address), aim (what we want to achieve) and scope (the service, group or population affected by the issue) of the pilot study was defined.

The study specifically targeted women aged 45–70 years (perimenopausal and postmenopausal stages) who use the services provided by the *Casa della Comunità* Le Piagge and are able to read and understand simple documents in the Italian language (scope and inclusion criteria). In this peculiar target group, the project focuses on the following modifiable determinants of cardiovascular health: HL, nutrition literacy, adherence to Mediterranean diet. The final aim of the pilot study is to give women knowledge and skills to increase their level of nutrition literacy and their adherence to Mediterranean Diet and will be reached in 2027.

The Ophelia process involves the use of the Health Literacy Questionnaire (HLQ) to measure HL, which uses cluster analysis to identify groups with similar HL profiles (see ‘Data analysis’). Sample size was not based on a conventional power calculation because no universally accepted formula exists for hierarchical clustering; adequacy depends on the number of indicators, the separation among clusters and the size of the smallest cluster. Within the HLQ/Ophelia framework, local surveys of 50–100 participants are typically considered adequate depending on the heterogeneity of the target group and a stable cluster analysis is generally achieved with approximately 80 complete HLQ questionnaires. Given the demographic characteristics of the participants to be included (relatively low variability) and based on other studies that have applied the Ophelia approach^23^,^27^ and therefore conducted cluster analyses based on HLQ responses—it is estimated that 150 women may be a sufficient number. Taking into account the possibility of some strongly incomplete questionnaires (which will need to be excluded from the cluster analysis), an oversampling of 30 women was considered.

Using a participatory approach, many stakeholders were involved in the project set-up and in the development of the study protocol, namely the social and healthcare workers of the *Casa della Comunità* Le Piagge, representatives from the LHU Tuscany Centre and from the municipality of Florence (policy makers), some non-governmental organisations (NGOs) working in Le Piagge, as well as the University of Florence (project leader). Stakeholders were involved both in study set-up as well as in the following steps and phases.

#### Local data collection (October 2024–January 2025)

Multiple strategies were adopted to inform the community about the study and to ensure inclusivity and representativeness. These included:

- collaboration with local stakeholders, including the University of Florence, the *Casa della Comunità* Le Piagge and community organisations (NGOs, citizens’ associations);
- snowball sampling to leverage community networks;
- offering rewards for participation (a plant of aromatic herbs).

**Figure 1:**
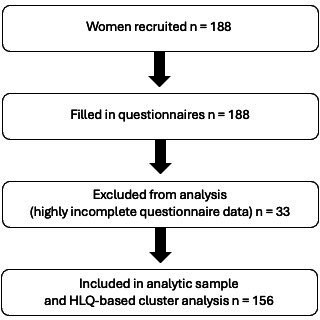
Flow of participants through recruitment and inclusion in the analytical sample. HLQ, Health Literacy Questionnaire.

Participants were recruited in the *Casa della Comunità* Le Piagge (figure 1). On designated days, researchers from the University of Florence were at the *Casa della Comunità* Le Piagge to recruit participants and collect data. Recruitment involved both women who voluntarily presented themselves to participate in the study and women in the waiting areas of the *Casa della Comunità* in the days devoted to the recruitment.

The questionnaires were self-completed on site, in the presence of the researchers, and collected immediately after completion. Recruiters were trained to adopt a standardised approach when inviting women to participate in the study and when providing support during questionnaire completion.

### Study instruments

Participants were invited to self-complete four questionnaires (see online supplemental table 1 for details):

- Sociodemographic and health-related questionnaire: to collect data on age, socioeconomic characteristics, health status, weight and height, smoking habit, physical activity. Marital status, medication burden and more detailed healthcare utilisation measures were not collected.^28^
- Sense of Coherence (SoC): SoC was assessed using the Italian adaptation of a semantic differential scale, as described by Bonaccorsi et al. The instrument is based on nine bipolar adjective pairs covering the three dimensions of comprehensibility, manageability and meaningfulness.^28^ Each item is rated on a 7-point semantic differential scale, with higher scores indicating a stronger SoC. Subscale scores were calculated separately for the three dimensions. In the present study, SoC was considered as a psychosocial correlate to support the interpretation of HL profiles and was not modelled as a mediator.
- HLQ: a validated 44-item instrument covering nine dimensions of HL. It is a self-perceived measurement tool with Likert-type items. The questionnaire is structured in two parts: scales 1–5 have a score range from 1 to 4, while scales 6–9 have a score range from 1 to 5. Scores for each scale are calculated as the mean of the corresponding items, with higher scores indicating greater HL strengths in each dimension. Data from the HLQ were analysed using cluster analysis to identify HL profiles.^18^ The nine HLQ scales are: (1) feeling understood and supported by healthcare providers, (2) having sufficient information to manage my health, (3) actively managing my health, (4) social support for health, (5) appraisal of health information, (6) ability to actively engage with healthcare providers, (7) navigating the healthcare system, (8) ability to find good health information and (9) understanding health information well enough to know what to do.
- Nutrition Literacy Instrument (NLit-IT): it measures functional, interactive and critical nutrition literacy. It is a performance-based tool whose final score ranged from 0 to 64. According to the final score, three levels of NL are identified: likelihood of poor NL (score ≤44), possibility of poor NL (score 45–57) and possibility of good NL (score ≥58).^29 30^
- MEDI-LITE: to assess the adherence to the Mediterranean diet, based on the frequency of self-reported consumption of nine food categories.^31^ The final score varies from 0 to 18. Scores between 0 and 5 indicated scarce adherence, between 6 and 12 medium adherence, and between 13 and 18 high adherence. With respect to other scales or tools to measure the adherence to the Mediterranean diet, the MEDI-LITE was chosen because it is particularly simple and quick, and suitable for measuring dietary behaviours consistent with the Mediterranean diet, even in populations not strictly from the Mediterranean area. No specific cultural or practical adaptation of the MEDI-LITE was required, as the instrument had already been validated for use in the Italian population.

The nine HLQ scales, the NLit-IT as well as the MEDI-LITE reported good reliability (online supplemental table 2).

### Statistical analysis

All the analyses were conducted using IBM SPSS V.28.0. A p value of 0.05 was considered as significant.

#### Missing data handling

For the cluster analysis, missing values in HLQ items were imputed when a maximum of 2 missing values for scales with 4–5 items or 3 missing values for scales with 6 items were observed; on the contrary, the responses were not included in the results for that participant on that scale. Specifically, the Expectation Maximisation algorithm was used for imputation.

#### Clustering approach

Cluster analysis was performed on the nine HLQ scale scores rather than on the 44 individual items, in accordance with the Ophelia approach.^18 19 32^ A hierarchical cluster analysis of cases was conducted using standardised z-scores, Ward’s method, and squared Euclidean distance as distance metric. To identify the optimal cluster solution, solutions ranging from 3 to 16 clusters were examined, within the range recommended in the Ophelia manual.^19^ Candidate solutions were evaluated based on the agglomeration schedule, within-cluster variability, and the interpretability and practical usefulness of the resulting HLQ profiles. In line with the Ophelia framework, cluster size was not used as a criterion for selecting the final solution, as smaller clusters may represent important vulnerable subgroups. In line with Ophelia guidance, within-cluster SDs were examined as an indicator of residual heterogeneity. Particular attention was also paid to the ability of the solution to identify clusters with comparatively disadvantaged HL profiles.

As a sensitivity analysis, non-hierarchical k-means clustering was also explored. The robustness of the selected solution was assessed through comparison of candidate solutions across methods, together with agglomeration-based inspection and evaluation of within-cluster variability.

To validate the selected cluster solution, both qualitative and quantitative criteria were considered. These included visual inspection of the dendrogram, examination of SDs within clusters, and profiling of clusters using the other variables collected in the study. This cluster analytic approach has been widely used in the Ophelia process and in other studies applying the HLQ.

Moreover, to assess whether the clustering solution was dependent on the missing-data handling strategy, two sensitivity analyses were performed. First, HLQ scale scores were recalculated using a within-scale mean imputation approach, applying the same predefined missingness thresholds as in the primary analysis. Specifically, scale scores were computed when the number of available items was sufficient according to the HLQ scoring rules used in the primary analysis; otherwise, the scale score was set to missing. Second, a complete-case clustering analysis was conducted among participants with no missing HLQ item data. For both sensitivity analyses, hierarchical cluster analysis was repeated using Ward’s method on standardised HLQ scale scores, and the resulting profiles were compared with the primary EM-based solution in terms of cluster number, cluster sizes and substantive interpretation of the HLQ patterns. The sensitivity analyses were not intended to replace the primary analysis, but to assess the robustness of the substantive interpretation of the profiles across reasonable missing-data strategies.

#### Statistical comparisons

Data are presented as mean (SD) and median (IQR) or percentages, as appropriate.

The scoring of each HLQ scale was calculated by averaging the item scores, with equal weighting.

To better profile the clusters, Fisher’s exact test and analysis of variance (ANOVA) were used to evaluate significant associations with categorical data and numerical data, respectively. Size effect was assessed using Cramer’s V for categorical variables and η² and ω² for continuous variables.

### Patient and public involvement

Patients and/or the public were not involved in the design, conduct, reporting or dissemination plans of this study. Participants were involved only as respondents to the questionnaires.

## Results

A total of 188 eligible women were recruited at the *Casa della Comunità* Le Piagge during the recruitment period and completed the study procedures after providing informed consent. For the HLQ-based cluster analysis, 32 participants were excluded because questionnaire completion was too incomplete to allow reliable inclusion, which exceeded the practical guidance for sample size in Ophelia (see Missing data handling). At the end, questionnaires filled in by 156 women were included in the analysis and described in this paper.

Participants’ age ranged from 47 to 70 years, with a mean of 58.50 (±6.2) years. The majority of participants (90.40%) were born in Italy, and most (79.50%) lived with their family members. Most participants were employed or retired, and the sample showed a relevant burden of chronic conditions and recent healthcare use, with 12.80% reporting at least one hospitalisation in the previous year.

Tables1  2 describe the main characteristics of the enrolled women.

**Table 1:** Main characteristics of the enrolled women—categorical variables (N=156)

Variables		N	%
Educational level	Less than high school diploma	39	25.00
	High school diploma	73	46.80
	Bachelor’s or master’s degree	44	28.20
Financial status: With the financial resources at your disposal—from your own or family income—how do you get to the end of the month?	Very easy	26	16.70
	Quite easy	55	35.30
	With some difficulties	62	39.70
	With many difficulties	8	5.10
	Missing		
Occupational status	Retired	40	25.60
	Employed	103	66.10
	Inactive	11	7.00
	Missing	2	1.30
Self-perceived health: How is your health in general?	Very good	9	5.50
	Good	45	28.80
	Fair	93	59.60
	Poor	7	4.50
	Very poor	1	0.60
	Missing	1	0.60
Hospitalisations in the past year	Yes	20	12.80
Chronic conditions	Arthritis	15	9.60
	Back pain	67	42.90
	Cardiovascular diseases	22	14.10
	Asthma or lung diseases	24	15.84
	Cancer	15	9.60
	Depression or anxiety	17	10.90
	Diabetes mellitus	11	7.10
	Stroke	3	1.90
	Thyroid dysfunction	13	8.30
Smoking habits	Active smoker	24	15.40
	Former smoker	54	34.60
	No smoker	77	49.40
	Missing	1	0.60
Nutritional status (BMI)	Underweight	6	3.80
	Healthy weight	65	41.70
	Overweight	54	34.60
	Obese	27	17.30
	Missing	4	2.60
Physical activity: In a typical week, on how many days do you engage in physical activity for a total of at least 30 min, causing at least a slight increase in breathing or heart rate, for example, playing sports or other leisure-time activities, at work, doing household chores or gardening, or travelling from one place to another?	Never	52	33.30
	Less than once a week–one a week	50	32.00
	2–3 times a week	30	19.20
	4–5 times a week	11	7.00
	6–7 times a week	11	7.00
	Don’t know/missing	2	1.30
Physical activity: Regarding physical activity and sports, how would you describe yourself?	Very active	13	8.30
	Quite active	60	38.50
	Not very active	54	34.60
	Sedentary	27	17.30
	Missing	2	1.30
Nutrition literacy (NLit-IT)	Likelihood of poor nutrition literacy	51	32.70
	Possibility of poor nutrition literacy	95	60.90
	Possibility of good nutrition literacy	4	2.60
	Missing	6	3.80
Adherence to Mediterranean Diet (MEDI-LITE)	Low	13	8.30
	Medium	87	55.80
	High	43	27.60
	Missing	13	8.30

BMI, body mass index; NLit-IT, Nutrition Literacy Instrument.

**Table 2:** Descriptive statistics of the scores at the Health Literacy Questionnaire by dimensions (N=156)

Scales	Theoretical score Range	Mean	SD	Minimum	Maximum	25th percentile	50th percentile	75th percentile
Feeling understood and supported by healthcare providers	1–4	3.02	0.58	1.50	4.00	2.75	3.00	3.25
Having sufficient information to manage my health	1–4	2.99	0.49	1.00	4.00	2.75	3.00	3.25
Actively managing my health	1–4	2.82	0.50	1.40	4.00	2.40	2.82	3.00
Social support for health	1–4	2.99	0.57	1.20	4.00	2.80	3.00	3.40
Appraisal of health information	1–4	2.96	0.48	1.60	4.00	2.60	3.00	3.20
Ability to actively engage with healthcare providers	1–5	3.60	0.71	1.40	5.00	3.00	3.60	4.00
Navigating the healthcare system	1–5	3.28	0.70	1.50	5.00	2.83	3.17	3.83
Ability to find good health information	1–5	3.44	0.69	1.80	5.00	3.00	3.60	3.96
Understand health information well enough to know what to do	1–5	3.65	0.67	2.00	5.00	3.20	3.60	4.00

The sample showed a mix of social and demographic backgrounds. Almost half of the women (46.80%) had completed secondary school, 28.20% had a university degree and one in four (25.00%) had not finished high school. Most were still working (66.10%), while 25.60% were retired and 7.00% were not working, showing that many were still active in the job market. Most women lived with family members, while a smaller proportion lived alone.

Financial status was generally modest. Just over half (52.00%) said they managed their monthly expenses easily or fairly easily, but many reported financial difficulties: 39.70% had some and 5.10% had serious difficulties.

Most participants rated their health as fair (59.60%), while 28.80% felt it was good and only 5.50% very good; 5.10% described it as poor or very poor. About 13% had been hospitalised at least once in the past year. The most common chronic problems were back pain (42.90%), respiratory diseases (15.80%), heart conditions (14.10%), depression or anxiety (10.90%), arthritis and cancer (both 9.60%). These data suggest that the cohort was characterised not only by social and economic variability but also by a non-negligible burden of chronic morbidity and healthcare needs.

Regarding lifestyle, about half of the women didn’t smoke (49.40%), 34.60% were former smokers and 15.40% still smoked. BMI data showed that 41.70% had a normal weight, 34.60% were overweight and 17.30% were obese. Physical activity levels were generally low: two out of three (65.30%) exercised once a week or less and only 14% did so at least four times per week.

NL was quite limited: 93.60% scored in the ‘poor’ or ‘possibly poor’ range and only 2.60% showed good knowledge. Mean and median values were low as well: 44.40 (±10.9) and 47 (IQR 40.5–52), respectively.

Based on MEDI-LITE categories, 55.80% of participants showed medium adherence to the Mediterranean diet, 27.60% high adherence and 8.30% low adherence; 8.30% had missing data. The mean MEDI-LITE score was 10.80 (SD 2.8) and the median was 11 (IQR 9–13).

The three dimensions of the SoC showed similar scores, with manageability presenting slightly lower mean and median scores (5.4 and 5.5, respectively)(table 3)(table 3).

**Table 3:** Main characteristics of the enrolled women—numerical variables

Variables	Score range	Mean (SD)	Median (IQR)
Sense of Coherence–comprehensibility	1–7	6.00 (1.1)	6.20 (5.2–7)
Sense of Coherence–manageability	1–7	5.40 (1.4)	5.50 (4.5–5.5)
Sense of Coherence–meaningfulness	1–7	5.90 (1.4)	6.30 (5.3–7.0)
Nutrition literacy (NLit-IT score)	0–64	44.40 (10.9)	47 (40.5–52)
Adherence to Mediterranean Diet (MEDI-LITE score)	0–18	10.80 (2.8)	11 (9–13)

NLit-IT, Nutrition Literacy Instrument.

The scores at the nine dimensions of the HLQ are described in table 2.

For HLQ scales 1–5, the final scores were generally around 3; the lowest score was observed for scale 3, ‘actively managing my health’ (2.82±0.50), while the highest score was observed for scale 1, ‘feeling understood and supported by healthcare providers’ (3.02±0.58). For HLQ scales 6–9, higher variability was observed; the lowest score was observed for scale 7, ‘navigating the healthcare system’ (3.28±0.70), while the highest score was observed for scale 9, ‘understand health information well enough to know what to do’ (3.65±0.67). These findings should be interpreted within the two distinct response ranges of the HLQ (1–4 for the scales 1–5, 1–5 for the scales 6–9) rather than by directly comparing all nine scales on a single metric.

A four-cluster solution was retained based on interpretability and between-cluster separation (ANOVA across domains, p<0.001).

For ease of interpretation, we assigned each cluster a descriptive functional label based on its overall HLQ pattern, while retaining the numerical cluster identifiers for transparency.

Regarding the cluster analysis, inspection of the agglomeration schedule indicated that the most plausible solutions were in the 3–4 cluster range, with clear increases in the agglomeration coefficient when moving from 4 to 3 clusters and, more markedly, from 3 to 2 clusters. Among these candidate solutions, the 4-cluster solution showed the lowest average within-cluster SD across the nine HLQ scales (0.34) compared with the 3-cluster (0.40) and 5-cluster (0.35) solutions, while maintaining an interpretable and meaningful cluster structure. The corresponding maximum within-cluster SD was also lowest for the 4-cluster solution (0.38 vs 0.50 for 3 clusters and 0.43 for 5 clusters). These findings suggested that the 4-cluster solution provided the best balance of internal homogeneity and practical interpretability. Comparison with k-means clustering for 3, 4 and 5-cluster solutions supports the choice of a 4-cluster solution as well. Sensitivity analyses were conducted to assess the robustness of the clustering solution to different missing-data strategies. The alternative within-scale mean imputation analysis included 154 participants and produced a four-cluster solution with cluster sizes of 15, 50, 62, and 27 participants. The resulting profiles showed a clear gradient across the nine HLQ domains, ranging from lower to higher HLQ scores, and were broadly consistent with the substantive interpretation of the primary EM-based solution. The complete-case analysis included 124 participants with no missing HLQ item data. This analysis also yielded an interpretable four-cluster solution, with cluster sizes of 9, 40, 52, and 23 participants. Although the exact cluster sizes differed from the primary analysis, the complete-case solution preserved the main distinction between HL profiles. Overall, these analyses suggested that the main interpretation of heterogeneous HL profiles was not materially driven by the EM imputation strategy, although exact cluster membership varied across missing-data approaches.

**Figure 2:**
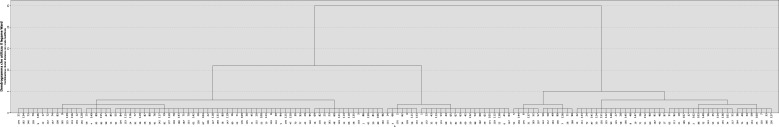
Dendrogram from Ward’s hierarchical clustering of HLQ scale scores (N=156). HLQ, Health Literacy Questionnaire.

The four distinct clusters represent different HL profiles within the sample, with statistically robust differentiation across all domains (ANOVA, p<0.001) (table 4, figure 2). Overall, the clusters displayed internally consistent patterns and a gradient in HL strengths and challenges across domains, reflecting the varied resources and needs of the women enrolled. On the other hand, for three scales (scale 4, ‘social support for health’, scale 5, ‘appraisal of health information’, scale 9, ‘understand health information well enough to know what to do’), the differences in scores across the four clusters were smaller than for the other six, indicating that women’s skill levels were more homogeneous in these domains.

**Table 4:** Cluster profiling: HLQ score by dimensions

Cluster ID		1–Broad HL strengths	2–Broad HL strengths with selective challenges	3–Widespread HL challenges	4–Mixed HL challenges with limited social support
**N (%**)		26 (16.7)	76 (48.7)	13 (8.3)	41 (26.3)
HLQ scales	Feeling understood and supported by healthcare providers (score range 1–4)	3.70 (0.32)	3.12 (0.39)	1.90 (0.26)	2.74 (0.38)
	Having sufficient information to manage my health (score range 1–4)	3.65 (0.29)	3.01 (0.24)	2.31 (0.40)	2.74 (0.46)
	Actively managing my health (score range 1–4)	3.32 (0.44)	2.87 (0.37)	2.03 (0.38)	2.63 (0.34)
	Social support for health (score range 1–4)	3.68 (0.32)	3.09 (0.33)	2.39 (0.66)	2.55 (0.45)
	Appraisal of health information (score range 1–4)	3.46 (0.38)	3.00 (0.39)	2.47 (0.39)	2.69 (0.39)
	Ability to actively engage with healthcare providers (score range 1–5)	4.47 (0.38)	3.73 (0.47)	2.40 (0.47)	3.20 (0.43)
	Navigating the healthcare system (score range 1–5)	4.21 (0.43)	3.39 (0.41)	2.24 (0.39)	2.79 (0.45)
	Ability to find good health information (score range 1–5)	4.22 (0.40)	3.53 (0.49)	2.43 (0.33)	3.07 (0.59)
	Understand health information well enough to know what to do (score range 1–5)	4.41 (0.38)	3.76 (0.46)	2.95 (0.73)	3.17 (0.50)

Colour shading is used to support visual interpretation of the HLQ profiles: green indicates relative strengths, yellow indicates intermediate scores and red indicates relative challenges within each HLQ scale.

For each dimension: p<0.001 (ANOVA).

ANOVA, analysis of variance; HL, health literacy; HLQ, Health Literacy Questionnaire.

Cluster 1 (broad HL strengths, n=26; 16.70%) showed the highest mean scores across all HLQ scales, indicating broad strengths across HLQ domains. Women in this group reported feeling well understood and supported by healthcare providers (scale 1; mean=3.70), having sufficient information to manage their health (scale 2; mean=3.65), demonstrating a strong ability to actively engage with providers (scale 6; mean=4.47), and to find and understand health information (scale 8, mean=4.22; scale 9, mean=4.41). This cluster represents participants with broad strengths in navigating the health system and in using health information effectively.

Cluster 2 (Broad HL strengths with selective challenges) included nearly half of the sample (n=76; 48.7%) and showed intermediate values across the HLQ scales (means ranging from 2.87 to 3.76). Scores on scales 1–5 suggested a generally positive profile, whereas scores on scales 6–9 reflected a more mixed pattern. In particular, values between 3.39 and 3.76 indicate that several HL tasks were not consistently easy, but rather fell between ‘sometimes difficult’ and ‘usually easy’. This cluster may therefore be described as having a mixed profile of HL strengths and challenges, depending on the domain. This cluster appears to represent women with generally adequate resources but with some more selective limitations across specific domains.

Cluster 3 (Widespread HL challenges, n=13; 8.30%) exhibited the lowest mean scores in almost all domains (means=1.90–2.95), indicating substantial challenges across the HLQ dimensions.

These women reported limited ability to find good health information (scale 8), low ability to critically appraise health information (scale 5), and poor perceived support from healthcare providers and social networks (scale 1 and scale 4). For scales 6–9, the observed scores indicate that several tasks were experienced as difficult, often closer to ‘usually difficult’ or ‘sometimes difficult’ than to ‘usually easy’. This profile points to a particularly vulnerable group in terms of HL resources.

Cluster 4 (mixed HL challenges with limited social support, n=41; 26.3%) showed scores slightly higher than cluster 3 (means=2.55–3.20) but still below the sample average. Participants in this group appeared to experience difficulties especially in scale 4, ‘social support for health’ (mean=2.55) and scale 5, ‘appraisal of health information’ (2.69). For scale 6, ‘ability to actively engage with healthcare providers’ and scale 8, ‘Ability to find good health information’ scores around 3.20 and 3.07 indicate that these tasks were only sometimes easy and still often difficult. This profile appears characterised not only by lower HL in several domains, but also by more limited social support.

Several variables were significantly associated with the four HL clusters, showing clear differences between social, health and psychosocial aspects (table 5). Effect size analysis showed that most between-cluster differences were small to moderate in magnitude. For categorical variables, Cramer’s V ranged from 0.20 to 0.25, indicating modest associations between cluster membership and variables such as living alone, educational level, financial status, self-perceived health, hospitalisations and adherence to the Mediterranean diet. Among these, adherence to the Mediterranean diet, self-perceived health and hospitalisations showed the largest effect sizes, although these remained below the threshold for a large effect. For continuous variables, SoC—meaningfulness displayed a large effect size (η²=0.198; ω²=0.178), suggesting that this dimension played a substantial role in differentiating the clusters.

**Table 5:** Cluster profiling: distribution of the variables significantly associated with clusters

Variables		Cluster				P value*	Effect size (95% CI)
		1 (N=26)	2 (N=76)	3 (N=13)	4 (N=41)		
Live alone	Yes	19.2%	17.10%	0	34.10%	0.03	0.23 (0.00 to 0.37) (p=0.035)
Sense of Coherence–meaningfulness		6.60 (1.00)	6.30 (1.00)	5.00 (1.90)	5.20 (1.50)	<0.001	η²=0.19 (0.08 to 0.29) ω²=0.17 (0.06 to 0.27)
Educational level	Less than high school diploma	34.60%	21.10%	23.10%	26.80%	0.03	0.20 (0.00 to 0.28) (p=0.05)
	High school diploma	19.20%	52.60%	38.50%	56.10%		
	Bachelor’s or master’s degree	46.20%	26.30%	38.50%	17.10%		
Financial status: With the financial resources at your disposal—from your own or family income—how do you get to the end of the month?	Very easy	30.40%	19.70%	0	9.80%	0.02	0.20 (0.02 to 0.26) (p=0.020)
	Quite easy	47.80%	38.20%	27.30%	29.30%		
	With some difficulties	21.70%	35.50%	54.50%	58.50%		
	With many difficulties	0	6.60%	18.20%	2.40%		
Self-perceived health: How is your health in general?	Very good	12.00%	7.90%	0	0	<0.001	0.24 (0.07 to 0.29) (p=0.006)
	Good	48.00%	32.90%	15.40%	14.60%		
	Fair	28.00%	57.90%	76.90%	78.00%		
	Poor	12%	1.30%	7.70%	4.90%		
	Very poor	0	0	0	2.40		
Hospitalisations in the past year	Yes	23.10%	3.90%	7.70%	24.40%	0.001	0.24 (0.09 to 0.43) (p=0.006)
Adherence to the Mediterranean Diet	Scarce	8.70%	8.20%	36.40%	2.80%	0.007	0.25 (0.09 to 0.34) (p=0.004)
	Medium	47.80%	64.40%	18.20%	75.00%		
	High	43.50%	27.40%	45.50%	22.20%		
Nutrition literacy (NLit-IT score)		44.70 (12.8)	46.00 (8.3)	35.90 (15.8)	43.90 (11.4)	0.029	η²=0.06 (0.001 to 0.13) ω²=0 (0.00 to 0.12)

For categorical variables: Cramer’s V; for continuous variable: η² and ω².

* Fisher’s exact test for categorical data, ANOVA for numerical data.

ANOVA, analysis of variance; NLit-IT, Nutrition Literacy Instrument.

Regarding education, women in clusters 1 and 2 were more often those with secondary or university education, whereas basic education was more common in clusters 3 and 4. A similar pattern emerged for financial situation: easier household finances were more frequent in clusters 1 and 2, while financial difficulties were more common in clusters 3 and 4, although these latter clusters should not be interpreted as uniformly disadvantaged across all indicators.

Perceptions of health followed a similar pattern. Good or very good health was more common in clusters 1 and 2, while fair or poor health was more frequent in clusters 3 and 4. However, hospital admissions did not follow a simple gradient, being slightly more frequent in clusters 1 and 4.

Lifestyle indicators also differed, but again not in a uniformly negative way for clusters 3 and 4. Clusters 1 and 2 showed the highest overall adherence to the Mediterranean diet when medium and high adherence were considered together, but cluster 4 still included a substantial proportion of women with medium adherence, and cluster 3 was not devoid of dietary resources, with a non-negligible proportion showing high adherence. NL showed a less favourable pattern overall, with NLit-IT scores lowest in cluster 3.

With regard to SoC, only the meaningfulness dimension was significantly associated with cluster membership (table 5), with higher mean values in clusters 1 and 2 and lower values in clusters 3 and 4. This finding suggests that the extent to which women perceived their life as meaningful may be related to their broader HL profile, while the present study was not designed to test directional or causal pathways.

Cluster interpretation was informed not only by HLQ patterns, but also by living arrangement, educational level, financial status, self-perceived health and hospitalisation in the past year.

## Discussion and conclusion

This study aimed to identify distinct HL profiles among women in the perimenopausal and postmenopausal period of life, living in a socioeconomically disadvantaged urban area of Florence, as part of a pilot study aimed at guiding cardiovascular prevention within the JACARDI project. As suggested by the Ophelia process, using the HLQ across its nine domains and complemented by measures of NL and adherence to the Mediterranean diet, we characterised patterns of HL strengths and weaknesses with the explicit aim of informing locally co-designed cardiovascular prevention initiatives.^33 34^ In doing so, the study provides granular evidence on how HL resources and needs are distributed in this population, offering a basis for subsequent co-design activities. This approach extends previous work on women’s cardiovascular HL, Mediterranean diet in menopause, and HL profiling by bringing these dimensions together within a single community-based sample.^6 32^

The analysis revealed four differentiated HL profiles with distinct patterns of strengths and challenges across domains. At one end, a smaller cluster showed high and relatively homogeneous HL across domains, while at the other, another cluster displayed more marked challenges across multiple domains, particularly in active self-management and critical appraisal. Between these poles, two mixed profiles emerged in which specific assets coexisted with deficits, for instance, relatively better communication with providers in the presence of weak navigation skills or adequate access to information alongside limited ability to act on it. Importantly, clusters 3 and 4 should not be viewed as uniformly deprived across all health-related indicators since some intermediate or preserved resources were also evident, particularly in relation to hospitalisation patterns and Mediterranean diet adherence. This multidimensional pattern underscores the value of profiling HL by domain rather than relying on a single summary score and is consistent with previous HL profiling approaches used to identify population subgroups with different patterns of strengths and challenges.^32 33^

Consistent associations were observed between cluster membership and key socioeconomic, behavioural and psychosocial variables. More marked HL challenges concentrated among women with lower education, financial strain and less favourable self-perceived health, whereas broader HL strengths co-occurred with higher educational attainment, easier household finances and better health perception. These patterns are consistent with a broad conceptualisation of HL as being shaped by social and contextual factors, embedded within and shaped by the social structure and resource distribution.^8 33^ In practical terms, the data suggest that social and contextual factors are relevant for interpreting HL profiles and should be considered during subsequent co-design activities.

Behavioural patterns tracked the same gradient: women in clusters with broader HL strengths were more often already practising and maintaining healthy behaviours, whereas those in clusters with more marked HL challenges were more often not yet changing their habits or only beginning to consider it. Differences also emerged in diet-related outcomes: adherence to the Mediterranean diet and NL were higher in clusters with stronger HL and lowest in the most disadvantaged cluster, echoing previous findings linking better HL with healthier diet adoption in vulnerable settings.^35^ Psychosocial resources followed a similar pattern: the meaningfulness dimension of SoC was higher in the upper-HL clusters and lower in the more disadvantaged clusters. In the present study, SoC was considered a psychosocial correlate that helped interpret the broader pattern of HL strengths and challenges and a useful characteristic to guide the following Ophelia phase (ie, the co-design of HL actions). Taken together, these results fit well with an integrated HL model that involves not only functional skills, but also interactive and critical competencies required to access, understand, appraise and apply information for care, prevention and health promotion.^8 36^ The observed gap between understanding information and enacting it in daily life, manifest in lower scores on domains related to active management and appraisal despite comparatively better performance in comprehension and access, speaks to the importance of strengthening the more ‘critical’ and ‘interactive’ layers of HL.^36^ In other words, knowing what to do is not always matched by the capacity, confidence or context to do it and interventions that focus solely on information provision are unlikely to close this gap. These profiles can also be read as short practical portraits. Women in the higher-HL clusters appeared more likely to combine informational, behavioural and psychosocial resources that may support engagement with prevention. By contrast, the lower-HL clusters, particularly the group with limited social support, point to women who may need more than information alone: simpler communication, help with service navigation, reinforcement over time and relational support may all be important for effective intervention planning.

Within the Ophelia framework, these profiles may guide differentiated and locally feasible actions. Higher-HL clusters may benefit mainly from reinforcement of existing preventive behaviours, whereas intermediate profiles may require targeted support in specific domains, such as communication or dietary counselling. Lower-HL clusters, particularly those with limited social support, may require more intensive approaches, including simplified communication, navigation support, closer follow-up and community-based support. These profile-informed needs can then be discussed with local stakeholders in the subsequent co-design phases of the Ophelia process and translated into context-appropriate interventions.

The proposed intervention directions are interpretative and intended to support the subsequent co-design phase; the specific actions will be defined collaboratively with participants and stakeholders.

The implementation of profile-informed interventions requires organisational coordination and attention to possible barriers, including staff time, language or communication needs and differences in social support across profiles. The co-design process should include the full range of identified profiles in order to avoid privileging women who already have stronger HL resources.

The present analysis is limited to the quantitative profiling stage of the Ophelia process. The subsequent qualitative and co-design phases, aimed at exploring participants’ experiences and translating the identified profiles into locally appropriate intervention strategies, have been performed as well, but will be presented in a subsequent paper.

These findings should be interpreted in light of the specific social and healthcare context of the Le Piagge service catchment. While the exact distribution of HL profiles may be context-specific, the broader identification of distinct profiles within socioeconomically disadvantaged primary care populations may be transferable to other underserved urban settings. Multisite studies in different Italian and European contexts are needed to assess transferability of the approach and the external validity of the results.

Methodologically, the multidimensional HLQ proved instrumental for generating a diagnostic map of needs. By distinguishing nine complementary domains, from feeling understood by providers to navigating the system and using information, HLQ profiling pinpointed specific bottlenecks (eg, appraisal, navigation, active management) while preserving assets (eg, provider engagement) that can be leveraged.^33^ This kind of fine-grained mapping is precisely what the Ophelia approach translates into fit-for-purpose actions through local co-design.^34^ Prior community-based applications of Ophelia in disadvantaged female populations found that tailoring to local HL needs was associated with improvements in HL and health behaviours. The reported pathways of change such as autonomy, competence, relatedness and empowerment are directly relevant to the present context.^37^ Rather than a generic education programme, the present profiles suggest different entry points and modalities depending on which domains are weakest within each cluster.

These findings reinforce and extend evidence that uneven HL distribution tracks with education and socioeconomic status and may contribute to health inequalities, thereby warranting explicit consideration in public health planning.^8 33 38^ In our setting, the co-occurrence of lower HL, economic strain, lower scores on the meaningfulness dimension of SoC and less healthy behaviours highlights a composite vulnerability that routine prevention messages may not adequately address. Conversely, the higher-HL profile appears to bring together educational, behavioural and psychosocial resources that may support greater engagement with preventive actions.

Implications for cardiovascular prevention planning are primarily related to the subsequent co-design phase. First, the data argue for a move beyond ‘one-size-fits-all’ information campaigns toward stratified, ‘profile-informed’ interventions. Possible examples to be explored during co-design include plain-language materials, visual supports, facilitated navigation, practical food education and peer-based support.^39^ When judging information is difficult, simple credibility checks, side-by-side comparisons of sources, and easy decision tools can support evaluation. When day-to-day self-management is weak, behavioural supports, for example, clear action plans, gentle reminders, or light peer check-ins, can help turn intentions into regular routines. Where NL is lowest, hands-on food education, for example, guided shopping and cooking sessions plus simplified Mediterranean diet guidance, can bridge understanding and everyday choices. In higher-HL clusters with narrower gaps, self-monitoring tools, light digital supports and peer-mentor roles can help maintain momentum and spread good practices across the community.

Second, the mode of delivery matters. Interventions that are co-designed from the ground up with women and local services as primary care teams, community organisations and neighbourhood associations are more likely to be acceptable, feasible and sustained. Subsequently, after having collected data, one focus group was conducted for each cluster with women belonging to that profile, to deepen barriers, resources and intervention priorities emerging from the HL profiling. In the next phase, we will implement targeted, context-specific interventions and actions co-designed with participants in each cluster - for example facilitated navigation, practical food education, self-management supports—with pre–post evaluation on relevant HLQ domains and behavioural outcomes. Co-creation processes surface lived experience and preferences, build trust where institutional confidence may be low, and adapt solutions to real-world constraints such as time, caregiving and transport.^17 24 25 27^ In practical terms, local ‘health labs’ for informal education and mutual-aid groups where women with stronger HL act as facilitators for peers can simultaneously address information, motivation and social support. Within Ophelia-style processes, the profiles presented here can be used to co-prioritise domains (eg, appraisal, navigation, active management), select delivery modes (eg, facilitated navigation, practical diet education, peer mentoring) and set realistic, measurable targets, such as incremental gains in the weaker HLQ domains and corresponding changes in diet adherence or activity stages.^36^

Third, policy should recognise HL as a strategic component of cardiovascular prevention. Embedding HL development in prevention plans, for example, community courses, provider training in clear communication, systematic use of plain-language materials, can increase reach and effectiveness at scale.^8^ Routine measurement of HL (and NL) in community assessments would enable programmes to target resources where the HL gap is widest, while monitoring change over time. In vulnerable urban areas, investing in HL, particularly in lower-HL clusters, can plausibly yield population benefits through better engagement, more equitable access and healthier behaviours.

The study offers several strengths. It applies a validated, multidimensional measure HLQ to profile HL in a clearly defined, socioeconomically disadvantaged female population, integrates NL and dietary patterns to anchor findings in behaviourally relevant outcomes, and employs cluster analysis to move beyond average effects toward actionable subgroups. By situating results within a co-designable framework, it bridges measurement and implementation, increasing the translational value for local services.

Some limitations merit consideration. The study focuses on one urban area and a specific age-sex group, so generalisability to other contexts may be limited. The voluntary nature of participation and recruitment in outpatient waiting rooms may have introduced a potential self-selection bias, with more health-conscious individuals being overrepresented. Recruiting women in a healthcare setting may have led to an underrepresentation of women with very low navigation HL and health information-seeking skills. On the other hand, the setting we considered is characterised by the wide range of primary care services it offers—from general practice to the blood sampling centre, from maternal and child health services to administrative offices for healthcare-related procedures—and it is well known to the local population, serving as a point of reference for numerous initiatives that are not only strictly healthcare-related but also social and health-related more broadly. Therefore, although the presence of selection bias cannot be definitively ruled out, we believe that in our study such bias is likely limited to peculiar groups, for whom studies with a different design would be needed.

Self-reported measures (eg, HL, health perception, behaviours) carry the usual risks of recall and social desirability bias. Regarding the first (recall bias) it is important to know that the scale and the tool we used referred to recent time periods, so reducing the recall bias. Regarding the latter, self-perceived measure of HL may lead to either overestimation or underestimation of individual HL skills, and that these tendencies vary according to socio-economic characteristics. Nevertheless, instruments assessing self-perceived HL skills are currently preferred over objective measures because they are better able to: (1) capture the multiple dimensions of the HL concept and (2) assess HL skills in relation to people’s life contexts, thereby reflecting HL as a relational construct. More generally, the use of items and scales already widely used and validated in different contexts resulted in limiting the information and the measurement bias.

Moreover, because cluster solutions are influenced not only by sample size but also by indicator choice, cluster separation, and the size of the smallest cluster, the four-cluster solution should be interpreted as exploratory and may require replication in an independent sample to establish its stability. These caveats do not diminish the practical value of the profiles for guiding local action but underscore the need for prospective evaluation. This was a cross-sectional, descriptive study and was not designed to support causal inference. The observed associations should therefore be interpreted as descriptive and hypothesis-generating. At the same time, they are likely to reflect bidirectional and reinforcing processes: poorer health may reduce the ability to engage with health information and preventive behaviours, while lower engagement may in turn contribute to less healthy behaviours and worse health outcomes. Future longitudinal studies should examine these reciprocal relationships over time, including the role of psychosocial resources in shaping them.

Finally, although sensitivity analyses using within-scale mean imputation and complete-case clustering supported the overall interpretation of heterogeneous HL profiles and a consistent gradient across HLQ domains, exact cluster sizes and membership varied across missing-data strategies. Therefore, the four-cluster solution should be interpreted as exploratory and as a practical, profile-informed tool for local intervention planning within the Ophelia framework, rather than as a definitive latent classification.

Future work should test whether profile-informed, co-designed actions to improve HLQ domain scores and, crucially, translate into behavioural and clinical gains relevant to cardiovascular prevention: diet quality, physical activity, blood pressure, lipids and long-term risk. It will also be important to examine cost-effectiveness and scalability in routine services, and to assess whether narrowing HL gaps narrows gradients in preventive uptake and outcomes. Incorporating mixed-methods evaluations could illuminate mechanisms, explaining how changes in comprehension, appraisal or navigation cascade into action and maintenance.

In conclusion, four HL profiles with a strong gradient and coherent socioeconomic, behavioural and psychosocial correlates were identified among perimenopausal and postmenopausal women in a disadvantaged urban setting. These findings support HL as a multifaceted construct and a meaningful lever for equity-oriented cardiovascular prevention. By pairing multidimensional measurement with community co-design, programmes can move beyond one-size-fits-all messaging toward targeted, acceptable and sustainable actions. These profiles can support the next Ophelia phases by helping participants, professionals and local stakeholders identify priorities for locally appropriate cardiovascular prevention actions.

## Supplementary material

10.1136/bmjopen-2025-115177 online supplemental file 1

## Data Availability

Data are available on reasonable request.

## References

[1] Mensah GA, Fuster V, Murray CJL (2023). Global Burden of Cardiovascular Diseases and Risks, 1990-2022. J Am Coll Cardiol.

[2] World Health Organization (2023). Cardiovascular diseases (CVDS). https://www.who.int/news-room/fact-sheets/detail/cardiovascular-diseases-(cvds).

[3] OECD/European (2022). Union. health at a glance: Europe 2022 – state of health in the EU cycle. https://health.ec.europa.eu/system/files/2022-12/2022_healthatglance_rep_en_0.pdf.

[4] Delanerolle G, Phiri P, Elneil S (2025). Menopause: a global health and wellbeing issue that needs urgent attention. Lancet Glob Health.

[5] Laffond A, Rivera-Picón C, Rodríguez-Muñoz PM (2023). Mediterranean Diet for Primary and Secondary Prevention of Cardiovascular Disease and Mortality: An Updated Systematic Review. Nutrients.

[6] Cano A, Marshall S, Zolfaroli I (2020). The Mediterranean diet and menopausal health: An EMAS position statement. Maturitas.

[7] Godos J (2023). Decreasing adherence to the Mediterranean diet: health and environmental foe. Int J Food Sci Nutr.

[8] Sørensen K, Van den Broucke S, Fullam J (2012). Health literacy and public health: a systematic review and integration of definitions and models. BMC Public Health.

[9] Berkman ND, Sheridan SL, Donahue KE (2011). Low health literacy and health outcomes: an updated systematic review. Ann Intern Med.

[10] Zanobini P, Lorini C, Lastrucci V (2021). Health Literacy, Socio-Economic Determinants, and Healthy Behaviours: Results from a Large Representative Sample of Tuscany Region, Italy. Int J Environ Res Public Health.

[11] Vettori V, Lorini C, Milani C (2019). Towards the Implementation of a Conceptual Framework of Food and Nutrition Literacy: Providing Healthy Eating for the Population. Int J Environ Res Public Health.

[12] Armocida B, Klepp K-I, Onder G (2025). Advancing Europe’s non-communicable diseases agenda through cross-national collaboration: translating WHO-Europe findings into actionable strategies. *Lancet Reg Health Eur*.

[13] Armocida B, Formenti B, Silano M (2024). Tackling the challenge of cardiovascular diseases and diabetes across Europe: a joint action by more than 300 public health professionals. Commentary. *Ann Ist Super Sanita*.

[14] Osborne RH, Elmer S, Hawkins M (2022). Health literacy development is central to the prevention and control of non-communicable diseases. BMJ Glob Health.

[15] World Health Organization (2022). Health literacy development for the prevention and control of noncommunicable diseases: volume 4. https://www.who.int/publications/i/item/9789240055391.

[16] Kickbusch I, Pelikan J, Apfel F (2013). Health literacy: the solid facts. https://iris.who.int/bitstream/handle/10665/128703/e96854.pdf.

[17] Leask CF, Sandlund M, Skelton DA (2019). Framework, principles and recommendations for utilising participatory methodologies in the co-creation and evaluation of public health interventions. *Res Involv Engagem*.

[18] Osborne RH, Batterham RW, Elsworth GR (2013). The grounded psychometric development and initial validation of the Health Literacy Questionnaire (HLQ). BMC Public Health.

[19] Osborne RH, Elmer S, Hawkins M (2021). The Optimising Health Literacy and Access (Ophelia) process to plan and implement National Health Literacy Demonstration Projects. Centre for Global Health and Equity, School of Health Sciences.

[20] Maciocco G (2020). La Casa della salute delle Piagge. https://www.saluteinternazionale.info/2020/01/la-casa-della-salute-delle-piagge/.

[21] Milani C, Naldini G, Baggiani L (2023). How to promote changes in primary care? The Florentine experience of the House of Community. Front Public Health.

[22] Beauchamp A, Batterham RW, Dodson S (2017). Systematic development and implementation of interventions to OPtimise Health Literacy and Access (Ophelia). BMC Public Health.

[23] Cheng C, Elsworth GR, Osborne RH (2020). Co-designing eHealth and Equity Solutions: Application of the Ophelia (Optimizing Health Literacy and Access) Process. Front Public Health.

[24] Windgassen S, Moss-Morris R, Goldsmith K (2018). The importance of cluster analysis for enhancing clinical practice: an example from irritable bowel syndrome. *J Ment Health*.

[25] Jessup RL, Osborne RH, Buchbinder R (2018). Using co-design to develop interventions to address health literacy needs in a hospitalised population. BMC Health Serv Res.

[26] Beauchamp A, Mohebbi M, Cooper A (2020). The impact of translated reminder letters and phone calls on mammography screening booking rates: Two randomised controlled trials. PLoS One.

[27] Aaby A, Simonsen CB, Ryom K (2020). Improving Organizational Health Literacy Responsiveness in Cardiac Rehabilitation Using a Co-Design Methodology: Results from The Heart Skills Study. *Int J Environ Res Public Health*.

[28] Bonaccorsi G, Zanobini P, Cosma C (2023). Do demographic and socio-economic factors predict Sense of Coherence among university students?. Ann Ist Super Sanita.

[29] Gibbs HD, Ellerbeck EF, Gajewski B (2018). The Nutrition Literacy Assessment Instrument is a valid and reliable measure of nutrition literacy in adults with chronic disease. J Nutr Educ Behav.

[30] Vettori V, Lorini C, Gibbs HD (2021). The Nutrition Literacy Assessment Instrument for Italian subjects, NLit-IT: exploring validity and reliability. Int J Environ Res Public Health.

[31] Sofi F, Dinu M, Pagliai G (2017). Validation of a literature-based adherence score to Mediterranean diet: the MEDI-LITE score. Int J Food Sci Nutr.

[32] Cheng C, Elmer S, Batterham R (2024). Measuring health literacy to inform actions to address health inequities: a cluster analysis approach based on the Australian national health literacy survey. J Public Health (Oxf).

[33] Batterham RW, Buchbinder R, Beauchamp A (2014). The OPtimising HEalth LIterAcy (Ophelia) process: study protocol for using health literacy profiling and community engagement to create and implement health reform. BMC Public Health.

[34] Batterham RW, Hawkins M, Collins PA (2016). Health literacy: applying current concepts to improve health services and reduce health inequalities. Public Health.

[35] Duarte A, Martins J, Lopes C (2024). Health literacy and its association with the adoption of the Mediterranean diet: a cross-sectional study. Nutrients.

[36] Nutbeam D (2025). Health literacy as a public health goal: 25 years on. Health Promot Int.

[37] Elmer S, Bridgman H, Williams A (2017). Evaluation of a health literacy programme for chronic conditions. *Health Lit Res Pract*.

[38] Albus C (2019). Health literacy: do we have effective interventions to enhance it in socially disadvantaged people?. Eur J Prev Cardiol.

[39] Greenberg KL, Leiter E, Donchin M (2019). Cardiovascular health literacy and patient–physician communication intervention in women from disadvantaged communities. *Eur J Prev Cardiolog*.

