# Supplementary material for: Optimising Health Literacy and Access (Ophelia) in Florence, Italy: a cluster analysis to guide cardiovascular prevention among perimenopausal and postmenopausal women in a vulnerable urban setting
Source: BMJ Open. 2026 Jul 16;16(7):e115177. doi: 10.1136/bmjopen-2025-115177 (PMC13384121; doi:10.1136/bmjopen-2025-115177)
Supplement: online supplemental file 1 [file bmjopen-16-7-s001.docx]

**Supplementary Table 1.** Detailed description of the four questionnaires used to collect data.

| **Questionnaires** | **Description** |
| --- | --- |
| Sociodemographic and health-related questionnaire | *Socio-demographic section*: age; area of residence; country of birth; language spoken at home; household composition; educational level; occupation; economic condition (“With the financial resources at your disposal - from your own or family income - how do you get to the end of the month?”, with four possible - “very easily”, “quite easily”, “with some difficulties”, “with many difficulties”).  *Health-related behaviours*: smoking habits (active smoker; former smoker; no smoker); two questions on physical activity (1. “In a typical week, on how many days do you engage in physical activity for a total of at least 30 minutes, causing at least a slight increase in breathing or heart rate, e.g., playing sports or other leisure-time activities, at work, doing household chores or gardening, or traveling from one place to another?”, with 10 possible response options – “never”, “less than once a week”, “one a week”, “two times a week”, “three times a week”, “four times a week”, “five times a week”, “six times a week”, “seven times a week”, “don’t know”. 2. “Regarding physical activity and sports, how would you describe yourself?", with four possible response options – “very active”, “quite active”, “not very active”, “sedentary”); weight and height).  *Sense of Coherence (SoC):* to assess SoC, we used the Italian version of the semantic differential scale developed by Vogt et al. and adapted for life in general [28]. The instrument includes one introductory question (“How do you personally find your current life situation in general?”) and nine bipolar adjective pairs rated on a 7-point semantic differential scale, with higher scores indicating a stronger sense of coherence. The nine items cover the three core dimensions of SoC: comprehensibility (items 1, 3, 6, and 9), manageability (items 4 and 7), and meaningfulness (items 2, 5, and 8). For each dimension, a subscale score was calculated as the mean of the corresponding item scores. In the present study, SoC was used as a psychosocial variable to characterise the study population and to support interpretation of the identified health literacy profiles; it was not modelled as a mediator or as a confounder in formal causal analyses. |
| Health Literacy Questionnaire (HLQ) | It is a self-perceived measure of HL consisting in 44 items in nine domains/scales [18]. The first 5 scales, constituting part 1 of the HLQ, are scored on a 4-point, Likert-type response scale (“strongly disagree”, “disagree”, “agree”, “strongly agree”). The last four scales, constituting part 2, are scored on a 5-point response scale, where respondents rate the item levels by the difficulty in undertaking a task (“cannot do”, “usually difficult”, “sometimes difficult”, “usually easy”, “always easy”). To each response, a score is assigned as follow:   - “strongly disagree” as 1, “disagree” as 2, “agree” as 3, “strongly agree” as 4; - “cannot do” as 1, “usually difficult” as 2, “sometimes difficult” as 3, “usually easy” as 4, “always easy” as 5.   The HLQ does not provide one overall summative score. The scoring of the HLQ is 9 scale scores, calculated by averaging the item scores within each scale with equal weighting. For the first 5 scales, the final score ranged from 1 to 4, while for le last four scales it ranged from 1 to 5. The higher the scores, the higher the HL skills. |
| Nutrition Literacy Instrument (NLit-IT): | It is the Italian version of the Nutrition Literacy Instrument measures functional, interactive, and critical nutrition literacy. It is a performance-based tools that covers the following subscales: “Nutrition and Health”, that measures reading comprehension of specific food elements (10 items); “Energy Sources in Food”, that measures knowledge of the macronutrient sources in food (10 items); “Household Food Measurement”, that measures identification of recommended portions (9 items); “Food Label and Numeracy”, that measures the ability to apply information obtained from the nutrition facts panel (10 items); “Food Groups”, that measures ability to classify foods by nutrition category and includes a list of 16 items; and “Consumers Skills”, that measures the ability to navigate food products to make healthy food choices (9 items). One point was given for each correct answer and the final score was calculated by summing each single item’s score. According to the final score, three levels of nutrition literacy are identified: likelihood of poor nutrition literacy (score ≤ 44), possibility of poor nutrition literacy (score 45–57), and possibility of good nutrition literacy (score ≥ 58) [30]. |
| MEDI-LITE | It is a questionnaire assessing adherence to the Mediterranean diet [31]. Overall, it includes nine food categories: (1) fruit; (2) vegetables; (3) cereal grains; (4) legumes; (5) fish and fish products; (6) meat and meat products; (7) dairy products; (8) alcohol intake and (9) olive oil. For food groups typical of the Mediterranean diet (fruit, vegetables, cereals, legumes and fish), a value of 2 was assigned to the highest category of consumption, 1 for the middle category and 0 for the lowest category. Conversely, for food groups not typical of the Mediterranean diet (meat and meat products, dairy products), a value of 2 was assigned for the lowest category, 1 for the middle category and 0 for the highest category of consumption. For alcohol, the categories related to the alcohol unit (1 alcohol unit = 12 g of alcohol) were used, by giving two points to the middle category (1–2 alcohol units/d), 1 point to the lowest category (1 alcohol unit/d) and 0 point to the highest category of consumption. Finally, two points were assigned for regular use of olive oil, 1 point for frequent use and 0 point for occasional use. The final score was obtained by summing these values, and it varies from 0 (low adherence) to 18 (high adherence). Scores between 0 and 5 indicated scarce adherence, while between 6 and 12 point out medium adherence. Optimal adherence results were noted with scores between 13 and 18. In the present study, the score was considered both as a continuous variable and as a categorical indicator of low, medium, or high adherence. No specific cultural adaptation of the instrument was required. The instrument was included because adherence to the Mediterranean diet is a relevant behavioural component of cardiovascular prevention in peri- and postmenopausal women. |

**Supplementary Table 2**: Cronbach’s alpha for each HLQ subscale, and for NLit-IT and MEDI-LITE.

| **Scale** | **Cronbach’s alpha** |
| --- | --- |
| HLQ1 - Feeling understood and supported by healthcare providers | 0.862 |
| HLQ2 - Having sufficient information to manage my health | 0.829 |
| HLQ3 - Actively managing my health | 0.794 |
| HLQ4 - Social support for health | 0.833 |
| HLQ5 - Appraisal of health information | 0.687 |
| HLQ6 - Ability to actively engage with healthcare providers | 0.826 |
| HLQ7 - Navigating the healthcare system | 0.828 |
| HLQ8 - Ability to find good health information | 0.828 |
| HLQ9 - Understanding health information well enough to know what to do | 0.819 |
| NLit-IT | 0.851 |
| MEDI-LITE | 0.600 |
