# Supplementary material for: Optimising Health Literacy and Access (Ophelia) in Florence, Italy: a cluster analysis to guide cardiovascular prevention among perimenopausal and postmenopausal women in a vulnerable urban setting
Source: BMJ Open. 2026 Jul 16;16(7):e115177. doi: 10.1136/bmjopen-2025-115177 (PMC13384121; doi:10.1136/bmjopen-2025-115177)
Supplement: Supplementary file 2 [file bmjopen-2025-115177.reviewer_comments.pdf]

PEER REVIEW HISTORY

BMJ Open publishes all reviews undertaken for accepted manuscripts. Reviewers are asked to complete a checklist review form and are provided with free text boxes to elaborate on their assessment. These free text comments are reproduced below.

ARTICLE DETAILS

Title (Provisional)

Optimizing Health Literacy and Access (Ophelia) in Florence, Italy: A Cluster Analysis to Guide Cardiovascular Prevention among Peri- and Postmenopausal Women in a Vulnerable Urban Setting

Authors

Cosma, Claudia; Bonaccorsi, Guglielmo; Biagi, Claudia; Milani, Chiara; Naldini, Giulia; Buresta, Diletta; Cerini, Gabriele; Graziani, Alice; Del Riccio, Marco; Zanobini, Patrizio; Volpi, Michele; Gallinoro, Veronica; Baggiani, Lorenzo; Nerattini, Marco; Lorini, Chiara

---

VERSION 1 - REVIEW

**Reviewer**	**1**
**Name**	**Hany, Mohamed**
**Affiliation**	**Alexandria University**
**Date**	**19-Jan-2026**
**COI**	**None**

Thank you for the opportunity to review this manuscript

This study explores health literacy among peri- and postmenopausal women at a community clinic in Florence, Italy. Using the HLQ, hierarchical cluster analysis identified four literacy profiles, which related to nutrition literacy, diet adherence, and sense of coherence. Among 188 women aged 45–70, 156 had complete data, revealing clusters: high HL (16.7%), moderate-to-high HL (48.7%), very low HL (8.3%), and low-to-moderate HL with limited social support (26.3%). Cluster membership is associated with education, financial difficulties, self-rated health, nutrition literacy, and diet adherence, indicating varied needs within this vulnerable group. However, the manuscript needs greater methodological transparency, including sample size justification, cluster validation, handling of missing data, sensitivity analyses, discussion of bias, and STROBE reporting, to ensure the findings are stable and useful for intervention.

Here are my comments/suggestions:

1. The manuscript does not provide a power/sample-size rationale for identifying stable clusters from 9 HLQ subscales with a final analytic sample of 156. Because clustering is sensitive to dimensionality, expected cluster separation, and the intended number of clusters, readers cannot judge whether the four-cluster solution is likely to be stable versus sample-specific noise. At least, add a dedicated justification grounded in clustering/latent-class considerations and explicitly frame the analysis as exploratory if that is the intent; commonly cited guidance includes Milligan & Cooper and the "2^k" rule used in related latent class contexts (Formann), while also acknowledging these are heuristics rather than guarantees of adequacy. Milligan GW, Cooper MC. An Examination of Procedures for Determining the Number of Clusters in a Data Set. Psychometrika. 1985;50(2):159–179. DOI: 10.1007/BF02294245. Formann AK. Die Latent-Class-Analyse: Einführung in Theorie und Anwendung. Weinheim: Beltz; 1984 (ISBN: 3407546572).

2. The manuscript does not clearly state the distance metric (e.g., Euclidean/Manhattan/correlation-based), linkage method (e.g., Ward/average/complete), nor a transparent approach for selecting the number of clusters. More importantly, there is no demonstration of cluster quality and reproducibility (e.g., silhouette values, Calinski–Harabasz index, bootstrap resampling, split-sample validation). Because hierarchical clustering can shift materially with small data perturbations, you should report (i) the exact algorithmic choices, (ii) internal validity metrics, and (iii) robustness checks comparing plausible alternatives (3- vs 4- vs 5-cluster solutions, and at least one non-hierarchical approach such as k-means or model-based clustering).

3. While the manuscript notes rules permitting imputation (e.g., up to two missing items in 4–5-item scales, up to three missing items in 6-item scales), it does not state how imputation was performed (mean within-scale, EM, multiple imputation, etc.), the proportion of imputed values, or whether missingness was MCAR/MAR/MNAR. Because imputed HLQ subscale values influence the clustering base, you should report missingness patterns and then run sensitivity analyses comparing (a) primary imputation, (b) an alternative imputation strategy, and (c) complete-case clustering to show whether cluster structure and downstream associations are consistent.

4. For clustering-based manuscripts, readers need to see whether findings persist across reasonable variations: different distance/linkage combinations, alternative clustering families (hierarchical vs partitioning vs model-based), inclusion/exclusion of outliers, standardized vs non-standardized inputs, and different cluster-number solutions. Without these, the reported four-cluster structure may be an artifact of a single pipeline rather than a reproducible profile set.

5. Recruiting from a healthcare facility likely selects for women who are already health-seeking and may under-represent those with the lowest access/engagement (potentially the lowest literacy). Self-report instruments for literacy, diet adherence, and psychosocial constructs are also vulnerable to social desirability and differential comprehension. Add a

“Bias” subsection detailing plausible sources (selection, information, measurement), what you did to mitigate them (standardized administration, training/QA), and—crucially—how each bias might shift associations (direction/magnitude) rather than a generic limitation statement.

6. Please add a participant flow diagram and explicitly account for the 32/188 participants not included in the analysis (ineligibility, decline, or missingness). Also, clarify whether the cross-sectional work was conducted prospectively within the clinic workflow or retrospectively assembled, and provide recruitment/data-collection dates. STROBE cross-sectional guidance explicitly recommends transparent reporting of participant flow and key design/method elements; see the STROBE checklist (cross-sectional) hosted by the EQUATOR Network.

7. Between-cluster comparisons should consistently include effect sizes (e.g., Cohen’s d/η^2 for continuous outcomes; Cramer’s V for categorical variables) and 95% CIs, not just p-values. This is particularly important because cluster sizes are uneven (e.g., the very low HL cluster is ~8.3%), making some comparisons underpowered and potentially unstable.

8. Education, income/financial difficulty, and age plausibly influence both cluster membership and secondary outcomes (nutrition literacy, diet adherence, sense of coherence). Present unadjusted comparisons, but also provide adjusted models (even if exploratory) to show whether cluster–outcome associations persist after key covariates. If adjustment is not feasible, justify why and explicitly reframe conclusions as descriptive/hypothesis-generating.

9. With many outcomes compared across four clusters, some statistically significant findings will occur by chance. Apply a correction strategy (e.g., false discovery rate) and report both unadjusted and adjusted p-values, interpreting “signals” more cautiously where they do not survive correction.

10. Even validated tools should have internal consistency reported in the study sample (e.g., Cronbach’s alpha for each HLQ subscale, and for NLit-IT and MEDI-LITE). Given the potentially lower educational level and greater vulnerability of the population, it is important to reassure readers that the scales performed reliably here.

11. The manuscript references an Italian adaptation of the Vogt et al. scale and highlights the “meaningfulness” difference but does not present full subscale results or clearly define whether sense of coherence is treated as an outcome, a mediator, or a confounder. Expand instrument details (items, scoring, psychometrics), present all relevant dimensions (comprehensibility/manageability/meaningfulness as applicable), and strengthen the conceptual model. If you propose it as a mediator between health literacy and behaviors, consider exploratory mediation analyses with appropriate caution.

12. MEDI-LITE scoring and interpretability need clearer reporting, providing the score range, how categories are interpreted (if any), and the distribution in this sample. Readers also

need a short justification for suitability in this specific vulnerable urban menopausal cohort and whether any cultural/practical adaptations were necessary.

13. A single neighborhood clinic in Florence is an important context, but it limits generalizability to other Italian/European settings with different social support structures and dietary patterns. Provide a transparent statement on which inferences are likely context-specific versus transferable, and outline what a multi-site replication would need to test.

14. Because Ophelia is typically a mixed-methods co-design process, readers will expect either (a) at least a summary of qualitative findings that informed interpretation/intervention planning, or (b) a clear statement that qualitative work is planned as a subsequent phase and is not part of the present report. If qualitative data exist, consider adding them (or, at a minimum, describing the methods and planned outputs).

15. For human-participant research, the manuscript should clearly state the ethics approval body, the approval number, the consent procedure, and the confidentiality/data protection measures.

16. Define "vulnerable" operationally with concrete neighborhood indicators. Because vulnerability is central to the rationale, include succinct area-level metrics (income, unemployment, education, deprivation index, housing indicators) for Le Piagge to help readers contextualize the cohort and assess transferability. Consider "socioeconomically disadvantaged," "high-deprivation urban area," or "underserved neighborhood," then define the operational indicators once (income/education/unemployment/deprivation index) so the term is evidence-based rather than impressionistic

17. The cluster labels are technically accurate but abstract; add 2–3-sentence portraits per cluster that translate HLQ patterns into practical realities (typical barriers, strengths, support needs), especially for the "very low HL" and "low-to-moderate HL with limited social support" groups.

18. Employment status, marital status, living arrangement details, comorbidities, medication burden, and healthcare utilization are either missing or unclear; these variables often shape both literacy and diet behavior and will help readers interpret cluster meaning.

19. Consider re-naming clusters with functionally meaningful descriptors (e.g., "Confident Navigators," "Resourceful Managers," "Isolated/Low-Support Strugglers") while still presenting the HLQ profile patterns transparently.

20. Because clustering is sensitive to scale and extreme values, specify whether HLQ subscales were z-standardized and how outliers were detected/handled; then show whether the solution persists when excluding influential points.

21. Expand the discussion beyond "cross-sectional limits causality" to explicitly note feedback loops (e.g., poorer health may reduce capacity to engage with information; disengagement may worsen diet), and propose a simple conceptual model that future longitudinal work could test.

22. The manuscript mentions cardiovascular prevention strategies, but readers will want clear examples of what would differ across clusters (communication style, navigation support, social prescribing, dietary coaching intensity, group vs. individual formats) and how the Ophelia co-design cycle would translate these profiles into locally feasible actions.

23. Discuss the resources required to implement tailored interventions in this clinic/community context, likely barriers (staff time, language/health-system complexity, social support gaps), and how the team will avoid inadvertently widening disparities (e.g., interventions that only benefit higher-literacy clusters).

24. Use “peri- and postmenopausal women” consistently (avoid switching to “menopausal women” unless defined). If peri- is included in eligibility, keep the phrasing consistent across title/abstract/methods/results to prevent misclassification in readers’ minds and in indexing.

25. Reorganize scattered methods details into a small number of clearly labeled subheadings. For example, keeping (i) participants/setting/dates, (ii) instruments, (iii) missing data, (iv) clustering pipeline and validation, and (v) statistical comparisons in distinct blocks will make the manuscript feel much more rigorous even before new analyses are added.

26. The Ophelia framework should be anchored with its core development paper when discussing co-design and implementation implications. Beauchamp A, Batterham RW, Dodson S, et al. Systematic development and implementation of interventions to OPtimise Health Literacy and Access (Ophelia). BMC Public Health. 2017;17:230. DOI: 10.1186/s12889-017-4147-5.

27. The novelty comparison references you listed are appropriate anchors for positioning (health literacy & CVD knowledge; women’s cardiovascular health literacy interventions; Mediterranean diet in menopause; clustering to identify vulnerable cardiovascular profiles; SDOH clustering; co-design with vulnerable populations). Citing these directly where you make comparative claims will strengthen the discussion’s credibility and reduce the risk of overclaiming uniqueness.

**Reviewer**	**2**
**Name**	**Cheng, Christina**
**Affiliation**	**La Trobe University**
**Date**	**21-Feb-2026**
**COI**	**None**

This is a generally well-written and clearly presented manuscript. The Introduction provides a solid foundation for the study. The Methods and Results are clearly presented. However, I have a few major concerns around the cluster solutions, the way health literacy is described and the interpretation of the HLQ scores.

Major comments:

1. According to the Ophelia manual p. 42, it indicates that the number of clusters are usually expected to be from 6 to 12 clusters. The manual indicates that the decision is more than just a quantitative decision but can also be a qualitative decision. The manual also indicates that sometimes clusters with similar patterns can be included due to the possibility that these clusters may have different demographic profiles, which means these clusters may need different interventions. Therefore, have you considered other cluster solutions to make sure that you are not somehow 'excluding' certain clusters with a disadvantaged profile?

2. The Ophelia process prefers to describe people as having health literacy strengths (higher scores) or challenges (lower scores) instead of describing people's health literacy as high/low, adequate/inadequate. With the 9 dimensions, the health literacy profiles, generated from the survey, allow you to consider interventions that take a strengths-based approach, i.e. consider what you can build on to support the domains indicating challenges. The Ophelia process tries to avoid labelling people as having high/low or adequate/inadequate health literacy.

3. The HLQ comprises of 2 parts, Part 1, i.e. Scales 1-5 has a score range of 1-4 while Part 2, i.e., Scale 6-9 has a score range of 1-5. Therefore, when presenting the HLQ results in the Results section, you cannot claim that Scales 9 and 6 has the 'highest' scores. The score of 2.99 for Scale 4 Social support for health is actually not 'modest', it is almost 3, which indicates participants generally agree that they have good social support. I'd say the score of 3.28 for Scale 7 Navigating the healthcare system is actually a little lower, as it's between a score of answering 'sometimes difficult' to answering 'usually easy' but would be closer to the 'sometimes difficult' response option. Or for Cluster 4, a score of 3 in Scales 6 and 8 means it's 'Sometimes difficult', not having good ability.

Given that you have compared the scale scores across different score ranges, I'd suggest you need to review your score interpretation for the full sample as well as for the four clusters and your Discussion section.

4. There is a tendency to describe Clusters 3 and 4 as being 'lower' than Clusters 1 and 2 in terms of the demographic characteristics. However, I see that the percentage of hospitalization is actually quite similar between Clusters 1 and 4. Cluster 4 has a pretty high proportion of people (75%) having medium adherence to the Mediterranean diet while Cluster 3 has close to half of people in this cluster being in high adherence. Need to consider that it's not doom and gloom when someone belongs to Clusters 3 and 4.

Minor comments:

5. Abstract – please provide more specific descriptions of health literacy instead of just high or low, see major comment above.

6. Strengths and limitations of this study – did you plan to conduct causal inference and you couldn't do it?

7. Introduction – the Ophelia process has 8 steps, not 9 steps. Phase 3 only has 2 steps.

8. Methods – p.44, line 53, 'foresees' may not be an appropriate word, can simply be 'uses'.

9. Methods – the description about the 9 scales/dimensions of the HLQ is missing, as well as the score ranges.

10. Methods – while you have the details of the other questionnaires in the supplementary file, you still need to provide the score interpretations in your Methods section, for easier references.

11. Results – 'financial status' is a better description than 'economic conditions'.

VERSION 1 - AUTHOR RESPONSE

Reviewer: 1
Dr. Mohamed Hany, Alexandria University

Comments to the Author:
Thank you for the opportunity to review this manuscript
This study explores health literacy among peri- and postmenopausal women at a community clinic in Florence, Italy. Using the HLQ, hierarchical cluster analysis identified four literacy profiles, which related to nutrition literacy, diet adherence, and sense of coherence. Among 188 women aged 45–70, 156 had complete data, revealing clusters: high HL (16.7%), moderate-to-high HL (48.7%), very low HL (8.3%), and low-to-moderate HL with limited social support (26.3%). Cluster membership is associated with education, financial difficulties, self-rated health, nutrition literacy, and diet adherence, indicating varied needs within this vulnerable group. However, the manuscript needs greater methodological transparency, including sample size justification, cluster validation, handling of missing data, sensitivity analyses, discussion of bias, and STROBE reporting, to ensure the findings are stable and useful for intervention.

Here are my comments/suggestions:

1-The manuscript does not provide a power/sample-size rationale for identifying stable clusters from 9 HLQ subscales with a final analytic sample of 156. Because clustering is sensitive to dimensionality, expected cluster separation, and the intended number of clusters, readers cannot judge whether the four-cluster solution is likely to be stable versus sample-specific noise. At least, add a dedicated justification grounded in clustering/latent-class considerations and explicitly frame the analysis as exploratory if that is the intent; commonly cited guidance includes Milligan & Cooper and the "2^k" rule used in related latent class contexts (Formann), while also acknowledging these are heuristics rather than guarantees of adequacy. Milligan GW, Cooper MC. An Examination of Procedures for Determining the Number of Clusters in a Data Set. Psychometrika. 1985;50(2):159–179. DOI: 10.1007/BF02294245. Formann AK. Die Latent-Class-Analyse: Einführung in Theorie und Anwendung. Weinheim: Beltz; 1984 (ISBN: 3407546572).
Response:

Thank you for this important comment. We agree that cluster-analytic solutions do not admit a conventional a priori power calculation in the same way as hypothesis-testing designs. We have therefore revised the manuscript to provide a dedicated sample-size rationale and to state more explicitly the exploratory intent of the analysis. In our study, clustering was conducted on the nine HLQ subscale scores, not on the 44 individual items, following the Ophelia procedure for HLQ data. Consistent with the Ophelia manual, we used hierarchical cluster analysis with Ward's method on standardized z-scores and compared alternative cluster solutions across the recommended range. The same manual indicates that, for a single target group, 50–100 respondents are often sufficient depending on heterogeneity, and that a stable cluster analysis is generally achieved with around 80 complete HLQ/eHLQ surveys. Our final analytic sample of 156 women with HLQ data therefore exceeded the practical guidance provided for HLQ/Ophelia clustering in a single, relatively homogeneous group.
At the same time, we now acknowledge explicitly that there is no universally accepted minimum sample-size rule for cluster- or latent-class-type analyses. Methodological work indicates that adequacy depends not only on sample size, but also on the number of indicators, the separation between clusters, and the relative size of the smallest cluster. Milligan and Cooper are highly relevant to the problem of cluster enumeration and the performance of stopping rules, but they do not provide a general minimum-N rule for hierarchical clustering. Likewise, heuristics such as Formann's 2^k rule arise from latent class contexts and, in our view, are not directly applicable to the present analysis, which used hierarchical clustering of continuous HLQ scale scores rather than model-based latent class analysis of categorical indicators. We have revised the Method and Limitation sections accordingly and specified that the four-cluster solution is exploratory and intended to identify potentially meaningful HL profiles, rather than as a definitive latent structure.

2-The manuscript does not clearly state the distance metric (e.g., Euclidean/ Manhattan/correlation-based), linkage method (e.g., Ward/average/complete), nor a transparent approach for selecting the number of clusters. More importantly, there is no demonstration of cluster quality and reproducibility (e.g., silhouette values, Calinski–Harabasz index, bootstrap resampling, split-sample validation). Because hierarchical clustering can shift materially with small data perturbations, you should report (i) the exact algorithmic choices, (ii) internal validity metrics, and (iii) robustness checks comparing plausible alternatives (3- vs 4- vs 5-cluster solutions, and at least one non-hierarchical approach such as k-means or model-based clustering).
Response:
Thank you for this important comment. We agree that the original manuscript did not describe the clustering procedure with sufficient transparency. We have now revised the Method as well as the Result sections to specify the exact analytic choices and to clarify the exploratory nature of the clustering.
As previusly detailed, cluster analysis was performed on the nine HLQ scale scores, not on the 44 individual items, following the Ophelia approach. In line with the Ophelia manual, we conducted hierarchical cluster analysis of cases using Ward's method on standardized z-scores, and examined cluster solutions from 3 to 16 clusters. Candidate solutions were evaluated using the agglomeration schedule, cluster sizes, profile interpretability, and within-cluster variability.

Inspection of the agglomeration schedule indicated that the most plausible solutions were in the 3- to 4-cluster range, with clear increases in the agglomeration coefficient when moving from 4 to 3 clusters and, more markedly, from 3 to 2 clusters. We therefore compared the 3-, 4-, and 5-cluster solutions in greater detail. Among these candidate solutions, the 4-cluster solution showed the lowest average within-cluster standard deviation across the nine HLQ scales (0.342) compared with the 3-cluster (0.409) and 5-cluster (0.351) solutions, while maintaining an interpretable minimum cluster size (n = 13). The corresponding maximum within-cluster SD was also lowest for the 4-cluster solution (0.385 vs 0.508 for 3 clusters and 0.430 for 5 clusters). These findings suggested that the 4-cluster solution provided the best balance of internal homogeneity and practical interpretability.
To assess robustness, we additionally compared the hierarchical results with k-means clustering for 3-, 4-, and 5-cluster solutions. Because silhouette statistics were not available in our SPSS configuration, we relied on the combination of agglomeration-based inspection, internal heterogeneity indices, and comparison across alternative clustering approaches. We have also revised the manuscript to frame the final 4-cluster solution as exploratory and hypothesis-generating, rather than as a definitive latent structure.

3-While the manuscript notes rules permitting imputation (e.g., up to two missing items in 4–5-item scales, up to three missing items in 6-item scales), it does not state how imputation was performed (mean within-scale, EM, multiple imputation, etc.), the proportion of imputed values, or whether missingness was MCAR/MAR/MNAR. Because imputed HLQ subscale values influence the clustering base, you should report missingness patterns and then run sensitivity analyses comparing (a) primary imputation, (b) an alternative imputation strategy, and (c) complete-case clustering to show whether cluster structure and downstream associations are consistent.
Response:
We thank the Reviewer for highlighting this lack of clarity. In the revised manuscript, we explicitly described how missing HLQ item values were handled according to the instrument scoring rules. Specifically, according to the HLQ scoring instructions, missing item responses was imputed using the provided Expectation Maximisation algorithm when they were within the allowable threshold (up to 2 missing items for 4-5-item scales and up to 3 missing items for 6-item scales); otherwise, the scale score was not computed for that respondent (that lead to 156 women included in the analysis).

4-For clustering-based manuscripts, readers need to see whether findings persist across reasonable variations: different distance/linkage combinations, alternative clustering families (hierarchical vs partitioning vs model-based), inclusion/exclusion of outliers, standardized vs non-standardized inputs, and different cluster-number solutions. Without these, the reported four-cluster structure may be an artifact of a single pipeline rather than a reproducible profile set.
Response:
Thanks for this comment. As previously described, the Method as well as the Results sections have been improved to increase clarity of the choice of the 4-cluster solution.

5- Recruiting from a healthcare facility likely selects for women who are already health-seeking and may under-represent those with the lowest access/engagement (potentially the lowest literacy). Self-report instruments for literacy, diet adherence, and psychosocial constructs are also vulnerable to social desirability and differential comprehension. Add a "Bias" subsection detailing plausible sources (selection, information, measurement), what you did to mitigate them (standardized administration, training/QA), and—crucially—how each bias might shift associations (direction/magnitude) rather than a generic limitation statement.
Response:

Thanks for the opportunity to clarify the impact of the study design on the collected data and the presented results. Details have also been added both in the Methods or in the Discussion Sections.
We acknowledge that recruiting women in a healthcare setting may have led to an underrepresentation of women with very low navigation health literacy and health information-seeking skills. On the other hand, the setting we considered is characterized by the wide range of primary care services it offers—from general practice to the blood sampling center, from maternal and child health services to administrative offices for healthcare-related procedures—and it is well known to the local population, serving as a point of reference for numerous initiatives that are not only strictly healthcare-related but also social and health-related more broadly. We also know from the international literature (es. the WHO M-POHL surveys) that people with lower levels of health literacy tend to use healthcare services more frequently, for example by visiting their general practitioner more often, albeit inappropriately. Therefore, although the presence of selection bias cannot be definitively ruled out, we believe that in our study such bias is likely limited to more "extreme" groups, for whom studies with a different design would be needed.
We also agree that self-reported measures (e.g., HL, health perception, behaviours) carry the usual risks of recall and social desirability bias. Regarding the first (recall bias) it is important to know that the scale and the tool we used referred to recent time periods, so reducing the recall bias. Regarding self-perceived measure of HL, they may lead to either overestimation or underestimation of individual HL skills, and that these tendencies vary according to socio-economic characteristics. Nevertheless, instruments assessing self-perceived HL skills are currently preferred over objective measures because they are better able to: (i) capture the multiple dimensions of the HL concept, and (ii) assess HL skills in relation to people's life contexts, thereby reflecting HL as a relational construct.
Finally, we would like to emphasize that each scale and each question used in our study was drawn from the international literature, has been validated in different contexts, and is widely used, including in national lifestyle surveillance systems.

6- Please add a participant flow diagram and explicitly account for the 32/188 participants not included in the analysis (ineligibility, decline, or missingness). Also, clarify whether the cross-sectional work was conducted prospectively within the clinic workflow or retrospectively assembled, and provide recruitment/data-collection dates. STROBE cross-sectional guidance explicitly recommends transparent reporting of participant flow and key design/method elements; see the STROBE checklist (cross-sectional) hosted by the EQUATOR Network.
Response:

In the revised manuscript, we added a participant flow diagram showing that 188 women were recruited and filled in the questionnaires, of whom 32 were excluded from the analytic sample because of highly incomplete questionnaire data that prevented reliable inclusion in the HLQ-based clustering. We also clarified that the study is a cross-sectional prospective data collection conducted within the ongoing Ophelia project, rather than a retrospective assembly of existing records. Moreover, we reported the recruitment/data-collection period more clearly and consistently in the Methods section. Finally, we added STROBE checklist to increase transparent reporting of the study.

7-Between-cluster comparisons should consistently include effect sizes (e.g., Cohen's d/η^2 for continuous outcomes; Cramer's V for categorical variables) and 95% CIs, not just p-values. This is particularly important because cluster sizes are uneven (e.g., the very low HL cluster is ~8.3%), making some comparisons underpowered and potentially unstable.
Response:
Effect sizes were added. On the contrary 95% Cis have not been added because the aim of this paper is to describe our cluster profiles to co-design fit-for-purpose HL actions, and not to generalize the results.

8-Education, income/financial difficulty, and age plausibly influence both cluster membership and secondary outcomes (nutrition literacy, diet adherence, sense of coherence). Present unadjusted comparisons, but also provide adjusted models (even if exploratory) to show whether cluster–outcome associations persist after key covariates. If adjustment is not feasible, justify why and explicitly reframe conclusions as descriptive/hypothesis-generating.
Response:
Thank you for this thoughtful comment. We agree that adjusted models may be useful when the aim is to estimate whether associations between cluster membership and external variables persist independently of key socio-demographic characteristics. However, this was not the purpose of the present study. Our analysis was conducted within the Ophelia framework, in which HLQ-based cluster analysis is used to identify meaningful health literacy profiles that can inform co-design of interventions with the target population. In this context, variables such as education, age, and financial difficulty are not viewed merely as confounders to be controlled for, but as part of the broader social and contextual characteristics that help define and interpret the profiles and their practical implications. The purpose of the comparisons with secondary variables was therefore descriptive, namely to characterize the identified clusters and to understand how they differed in terms of psychosocial, socio-economic, and health-related features relevant for intervention design. For this reason, we did not conduct adjusted models, as doing so would have addressed a different question—namely, whether cluster membership was associated with these variables independently of selected covariates—rather than the descriptive and practice-oriented objective of the Ophelia approach.

9-With many outcomes compared across four clusters, some statistically significant findings will occur by chance. Apply a correction strategy (e.g., false discovery rate) and report both unadjusted and adjusted p-values, interpreting "signals" more cautiously where they do not survive correction.
Response:

Thank you for this comment. We agree that, when multiple statistical comparisons are used in a confirmatory framework, some statistically significant findings may arise by chance and a correction strategy such as false discovery rate may be appropriate. However, this was not the purpose of the present analysis.
As previously described, in our study, comparisons across clusters on socio-demographic, psychosocial, and health-related variables were conducted to describe and characterize the identified HLQ profiles within the Ophelia framework, rather than to test a predefined set of confirmatory hypotheses. For this reason, we did not apply a formal multiple-comparison correction, as doing so would have shifted the interpretation toward a confirmatory inferential framework that was not the aim of the study.
To address the reviewer's concern, we have revised the manuscript to make the exploratory and hypothesis-generating nature of these comparisons explicit, and we now interpret the findings more cautiously, giving greater emphasis to the magnitude of effect sizes and the overall consistency of the profile patterns rather than to p-values alone.

10-Even validated tools should have internal consistency reported in the study sample (e.g., Cronbach's alpha for each HLQ subscale, and for NLit-IT and MEDI-LITE). Given the potentially lower educational level and greater vulnerability of the population, it is important to reassure readers that the scales performed reliably here.
Response: Cronbach's alpha for each HLQ subscale, and for NLit-IT and MEDI-LITE have been added (see Supplementary Table 2).

11-The manuscript references an Italian adaptation of the Vogt et al. scale and highlights the "meaningfulness" difference but does not present full subscale results or clearly define whether sense of coherence is treated as an outcome, a mediator, or a confounder. Expand instrument details (items, scoring, psychometrics), present all relevant dimensions (comprehensibility/manageability/meaningfulness as applicable), and strengthen the conceptual model. If you propose it as a mediator between health literacy and behaviors, consider exploratory mediation analyses with appropriate caution.
Response:
We thank the Reviewer for this suggestion. In the revised manuscript, we expanded the description of the Sense of Coherence instrument in the Methods section by clarifying the dimensions considered, the scoring approach, and the interpretive framework used in this study. We also strengthened the conceptual framing in the Introduction/Discussion by clarifying how sense of coherence is positioned in the present manuscript—namely as a psychosocial correlate relevant to the interpretation of the health literacy profiles rather than as a formally tested mediator. Please, note that the aim of this paper is to describe the results of the first two steps of the Ophelia process, that are those devoted to the quantitative evaluation of HL, knowledge and behavioural needs of the target group. In this perspective, the Sense of Coherence has to be considered as one of the characteristics of the women relevant to describe the profiles – as for age, educational level and economic resources – and to drive the following Ophelia steps.

12-MEDI-LITE scoring and interpretability need clearer reporting, providing the score range, how categories are interpreted (if any), and the distribution in this sample. Readers also need a short justification for suitability in this specific vulnerable urban menopausal cohort and whether any cultural/practical adaptations were necessary.

Response:
We agree. In the revised manuscript, we provided a clearer description of the MEDI-LITE score range and category interpretation directly in the Methods section, rather than relying mainly on supplementary material. We also detailed that the distribution of MEDI-LITE scores in the Results. Moreover, we added a brief justification for the relevance of this instrument in the context of cardiovascular prevention among peri- and postmenopausal women in this urban vulnerable setting. No specific cultural adaptation of the instrument was applied – since it was already validated in the Italian language for Italian people - and we clarified this point where appropriate.

13-A single neighborhood clinic in Florence is an important context, but it limits generalizability to other Italian/European settings with different social support structures and dietary patterns. Provide a transparent statement on which inferences are likely context-specific versus transferable, and outline what a multi-site replication would need to test.
Response:
We agree and will strengthen this point in the Discussion. In the revised version, we made a clearer distinction between findings that are likely to be context-specific to the Le Piagge service catchment and findings that may be transferable to other underserved urban settings with similar social and healthcare access conditions. We also noted more explicitly that broader external validity would require multi-site replication across different Italian and European contexts.
Nevertheless, we would like to emphasize the key elements and underlying principles of the Ophelia process. This process was originally developed with the aim of providing a systematic methodology for health literacy development that is grounded in local needs and in the co-design of context-specific actions with the target population (i.e., a fit-for-purpose approach). Notably, these are also the core principles underpinning health promotion interventions developed for and with communities. From this perspective, the profiles (clusters) identified in our study reflect the characteristics of the local target population and are therefore not necessarily generalizable to other settings. What may be considered generalizable, however, is the Ophelia methodology itself.

14-Because Ophelia is typically a mixed-methods co-design process, readers will expect either (a) at least a summary of qualitative findings that informed interpretation/intervention planning, or (b) a clear statement that qualitative work is planned as a subsequent phase and is not part of the present report. If qualitative data exist, consider adding them (or, at a minimum, describing the methods and planned outputs).
Response:
We thank the Reviewer for this important clarification. In the revised manuscript, we stated more clearly that the present paper reports only the quantitative phase of the Ophelia process, corresponding to the initial profiling stage, and that the qualitative/co-design phases will be described in another paper. This clarification was be added both in the Methods and in the Discussion so that readers do not expect qualitative findings in the current manuscript.

15-For human-participant research, the manuscript should clearly state the ethics approval body, the approval number, the consent procedure, and the confidentiality/data protection measures.
Response:

In the revised manuscript, we replaced the previous ethics statement with a clearer dedicated statement in the Methods section. This statement reports the ethics approval body and approval number already cited in the paper, specifies that written informed consent was obtained before participation and questionnaire completion, and clarifies the confidentiality/data protection safeguards applied during data collection and management.

16-Define “vulnerable” operationally with concrete neighborhood indicators. Because vulnerability is central to the rationale, include succinct area-level metrics (income, unemployment, education, deprivation index, housing indicators) for Le Piagge to help readers contextualize the cohort and assess transferability. Consider “socioeconomically disadvantaged,” “high-deprivation urban area,” or “underserved neighborhood,” then define the operational indicators once (income/education/unemployment/deprivation index) so the term is evidence-based rather than impressionistic

Response:

We agree that the term should be operationalized more explicitly. In the revised manuscript, we replaced generic wording with a more evidence-based description of Le Piagge as a socioeconomically disadvantaged/high-deprivation urban area, and we anchored this description to the contextual indicators and references already cited for the neighbourhood. This will help readers understand the rationale for focusing on this setting and better assess the transferability of the findings.

17-The cluster labels are technically accurate but abstract; add 2–3-sentence portraits per cluster that translate HLQ patterns into practical realities (typical barriers, strengths, support needs), especially for the “very low HL” and “low-to-moderate HL with limited social support” groups.

Response:

In the revised Results and Discussion sections, we added short narrative “portraits” for each cluster to translate the HLQ patterns into more practical terms, including likely strengths, barriers, and support needs. We agree this is especially important for the clusters characterized by lower scores and limited social support, where a purely technical profile may not be sufficiently informative for intervention planning.

18-Employment status, marital status, living arrangement details, comorbidities, medication burden, and healthcare utilization are either missing or unclear; these variables often shape both literacy and diet behavior and will help readers interpret cluster meaning.

Response:

We thank the Reviewer for this comment. Several of these characteristics are already present in the descriptive tables (e.g., occupational status, living arrangement, chronic conditions, hospitalization in the past year), but we agree that they are not sufficiently highlighted in the narrative text. In the revised manuscript, we improved the Results section so that these characteristics are described more clearly and more consistently as part of the sample profile and cluster interpretation. We also made explicit, where relevant, which variables were collected and which were not available in the present study.

19-Consider re-naming clusters with functionally meaningful descriptors (e.g., “Confident Navigators,” “Resourceful Managers,” “Isolated/Low-Support Strugglers”) while still presenting the HLQ profile patterns transparently.

Response:

We thank the reviewer for this helpful suggestion. In the revised manuscript, we have introduced functionally meaningful descriptive labels for the four clusters, while retaining the original numerical cluster identifiers (Clusters 1–4) to preserve transparency and direct correspondence with the HLQ profile patterns. These labels are intended as interpretive aids only and were chosen to reflect the observed HLQ configuration without overstating the certainty or stability of the profiles.

20-Because clustering is sensitive to scale and extreme values, specify whether HLQ subscales were z-standardized and how outliers were detected/handled; then show whether the solution persists when excluding influential points.
Response:

As previously described, we have revised the Method as well as the Result sections to specify the cluster analysis.

21-Expand the discussion beyond "cross-sectional limits causality" to explicitly note feedback loops (e.g., poorer health may reduce capacity to engage with information; disengagement may worsen diet), and propose a simple conceptual model that future longitudinal work could test.
Response:
We agree. In the revised Discussion, we expanded the interpretation of the cross-sectional design by explicitly acknowledging the likely bidirectional and reinforcing relationships between health literacy, health status, psychosocial resources, and health behaviours. We also added a simple conceptual description of these possible feedback loops to better frame the implications for future longitudinal work.

22-The manuscript mentions cardiovascular prevention strategies, but readers will want clear examples of what would differ across clusters (communication style, navigation support, social prescribing, dietary coaching intensity, group vs. individual formats) and how the Ophelia co-design cycle would translate these profiles into locally feasible actions.
Response:
We thank the Reviewer for this constructive suggestion. In the revised Discussion, we provided more concrete examples of how the identified profiles could inform differentiated intervention strategies within the Ophelia framework, for example, varying communication support, navigation assistance, social support components, and intensity or format of dietary counselling according to cluster characteristics. This should help make the translational value of the profiling exercise more explicit. However, we would like to emphasize that, in the Ophelia process, and therefore also in our study, the selection of actions and interventions does not derive exclusively from this data collection phase. Rather, it is informed by the subsequent qualitative phase and by the idea-generation workshops involving the other relevant stakeholders (e.g., health and social care professionals, representatives of voluntary associations, and policy-makers), according to a co-design process.

23-Discuss the resources required to implement tailored interventions in this clinic/community context, likely barriers (staff time, language/health-system complexity, social support gaps), and how the team will avoid inadvertently widening disparities (e.g., interventions that only benefit higher-literacy clusters).

Response:
We thank the Reviewer for this important point. In the revised Discussion, we have clarified the implementation perspective in a way that is specific to our setting. In this project, data collection and intervention planning were supported by dedicated project staff, and the study procedures were conducted in Italian, consistent with the eligibility criteria of the present phase. We have therefore avoided overstating barriers that were not directly encountered in this context. At the same time, we now note that profile-informed interventions still require organizational coordination and attention to differential support needs across groups. We also clarify that, as already mentioned, within the Ophelia approach, the co-design process is intended to involve the full range of identified profiles, so that proposed actions are shaped not only around women with stronger resources, but also around those with lower support and more limited navigation capacity.

24-Use “peri- and postmenopausal women” consistently (avoid switching to “menopausal women” unless defined). If peri- is included in eligibility, keep the phrasing consistent across title/abstract/methods/results to prevent misclassification in readers’ minds and in indexing.
Response:
We agree and will revise the manuscript for consistency. The expression “peri- and postmenopausal women” is now used uniformly throughout the title, abstract, methods, results, and discussion to avoid ambiguity or unintended misclassification.

25-Reorganize scattered methods details into a small number of clearly labeled subheadings. For example, keeping (i) participants/setting/dates, (ii) instruments, (iii) missing data, (iv) clustering pipeline and validation, and (v) statistical comparisons in distinct blocks will make the manuscript feel much more rigorous even before new analyses are added.
Response:
We fully agree. In the revised manuscript, we reorganized the Methods section into clearer subsections, covering at minimum setting/participants/recruitment period; instruments; missing data handling; clustering approach; and statistical comparisons.
We agree that this restructuring substantially improves readability and perceived methodological rigor.

26-The Ophelia framework should be anchored with its core development paper when discussing co-design and implementation implications. Beauchamp A, Batterham RW, Dodson S, et al. Systematic development and implementation of interventions to OPtimise Health Literacy and Access (Ophelia). BMC Public Health. 2017;17:230. DOI: 10.1186/s12889-017-4147-5.
Response:
Thank you for this suggestion. We have added the recommended Beauchamp et al. reference in the manuscript where the Ophelia framework and its co-design and implementation principles are discussed.

27-The novelty comparison references you listed are appropriate anchors for positioning (health literacy & CVD knowledge; women’s cardiovascular health literacy interventions; Mediterranean diet in menopause; clustering to identify vulnerable cardiovascular profiles; SDOH clustering; co-design with vulnerable populations). Citing these directly where you

make comparative claims will strengthen the discussion's credibility and reduce the risk of overclaiming uniqueness.

Response:

We agree and have strengthened the Discussion by citing the relevant comparative references directly at the points where we position our findings in relation to previous literature. This helps place the present study more precisely within the existing evidence and reduces the risk of overstating its novelty.

Reviewer: 2

Dr. Christina Cheng, La Trobe University

Comments to the Author:

This is a generally well-written and clearly presented manuscript. The Introduction provides a solid foundation for the study. The Methods and Results are clearly presented. However, I have a few major concerns around the cluster solutions, the way health literacy is described and the interpretation of the HLQ scores.

Major comments:

1. According to the Ophelia manual p. 42, it indicates that the number of clusters are usually expected to be from 6 to 12 clusters. The manual indicates that the decision is more than just a quantitative decision but can also be a qualitative decision. The manual also indicates that sometimes clusters with similar patterns can be included due to the possibility that these clusters may have different demographic profiles, which means these clusters may need different interventions. Therefore, have you considered other cluster solutions to make sure that you are not somehow 'excluding' certain clusters with a disadvantaged profile?

   Response:

   Thanks for this comment. As suggested in the Ophelia manual, cluster analysis was performed on the nine HLQ scale scores, using the hierarchical cluster analysis approach of cases using Ward's method on standardized z-scores, and examined cluster solutions from 3 to 16 clusters. Candidate solutions were evaluated using the agglomeration schedule, cluster sizes, profile interpretability, and within-cluster variability. Inspection of the agglomeration schedule indicated that the most plausible solutions were in the 3- to 4-cluster range, with clear increases in the agglomeration coefficient when moving from 4 to 3 clusters and, more markedly, from 3 to 2 clusters. We therefore compared the 3-, 4-, and 5-cluster solutions in greater detail. Among these candidate solutions, the 4-cluster solution showed the lowest average within-cluster standard deviation across the nine HLQ scales (0.342) compared with the 3-cluster (0.409) and 5-cluster (0.351) solutions, while maintaining an interpretable minimum cluster size (n = 13). The corresponding maximum within-cluster SD was also lowest for the 4-cluster solution (0.385 vs 0.508 for 3 clusters and 0.430 for 5 clusters). These findings suggested that the 4-cluster solution provided the best balance of internal homogeneity and practical interpretability. To assess robustness, we additionally compared the hierarchical results with k-means clustering for 3-, 4-, and 5-cluster solutions. To summarize, we are aware that the Ophelia manual suggests

considering at least 6 clusters, but the analysis results seem to support the choice of a smaller number of clusters. Please, note that our target is well defined (women aged 45-70 years old, living in a specific area of Florence), so the presence of a low numeber of clusters was expected.

2. The Ophelia process prefers to describe people as having health literacy strengths (higher scores) or challenges (lower scores) instead of describing people's health literacy as high/low, adequate/inadequate. With the 9 dimensions, the health literacy profiles, generated from the survey, allow you to consider interventions that take a strengths-based approach, i.e. consider what you can build on to support the domains indicating challenges. The Ophelia process tries to avoid labelling people as having high/low or adequate/inadequate health literacy.
Response:
We thank the Reviewer for this important conceptual point. We agree that some of the current wording does not fully reflect the strengths-based orientation of the Ophelia process. In the revised manuscript, we will revise the language throughout the abstract, results, discussion, and cluster descriptions to avoid reductive labels such as "high/low" or "adequate/inadequate" health literacy where a more nuanced description of strengths and challenges across domains is more appropriate. We will also ensure that the cluster narratives highlight not only areas of challenge, but also domains that may represent strengths to build on in tailored intervention design. As also suggested by Reviewer 1 we have assigned a descriptive label to each cluster to facilitate clearer and more meaningful identification, while maintaining consistency with the underlying HLQ profile patterns.

3. The HLQ comprises of 2 parts, Part 1, i.e. Scales 1-5 has a score range of 1-4 while Part 2, i.e., Scale 6-9 has a score range of 1-5. Therefore, when presenting the HLQ results in the Results section, you cannot claim that Scales 9 and 6 has the 'highest' scores. The score of 2.99 for Scale 4 Social support for health is actually not 'modest', it is almost 3, which indicates participants generally agree that they have good social support. I'd say the score of 3.28 for Scale 7 Navigating the healthcare system is actually a little lower, as it's between a score of answering 'sometimes difficult' to answering 'usually easy' but would be closer to the 'sometimes difficult' response option. Or for Cluster 4, a score of 3 in Scales 6 and 8 means it's 'Sometimes difficult', not having good ability.
Given that you have compared the scale scores across different score ranges, I'd suggest you need to review your score interpretation for the full sample as well as for the four clusters and your Discussion section.
Response:
We fully agree and thank the Reviewer for identifying this issue. In the revised manuscript, we carefully revised the interpretation of HLQ scores to reflect the fact that scales 1-5 and scales 6-9 have different response ranges and should not be directly compared as if they were on the same metric. Accordingly, we removed wording that implies that one scale had the "highest" or "lowest" score across the full set of nine dimensions when such comparisons are not appropriate. We also revised cluster-level descriptions to align more closely with the actual response meaning of each scale, especially for scales 6-9, where scores near 3 indicate a level closer to "sometimes

difficult" than to a clearly high ability. More generally, we revised the Results and Discussion so that the interpretation of HLQ findings is more accurate and more faithful to the structure of the instrument.

4. There is a tendency to describe Clusters 3 and 4 as being 'lower' than Clusters 1 and 2 in terms of the demographic characteristics. However, I see that the percentage of hospitalization is actually quite similar between Clusters 1 and 4. Cluster 4 has a pretty high proportion of people (75%) having medium adherence to the Mediterranean diet while Cluster 3 has close to half of people in this cluster being in high adherence. Need to consider that it's not doom and gloom when someone belongs to Clusters 3 and 4.
   Response:
   We agree. In the revised manuscript, we made the interpretation of Clusters 3 and 4 more balanced and nuanced. In particular, we avoided language that may portray these groups as uniformly disadvantaged across all domains and will acknowledge the features that suggest preserved or intermediate resources, for example, the non-negligible proportion with medium dietary adherence in Cluster 4 and the not uniformly worse profile on all health-related indicators. This change was also consistent with the strengths-based perspective emphasized in the Ophelia approach.

Minor comments:

5. Abstract – please provide more specific descriptions of health literacy instead of just high or low, see major comment above.
   Response:
   We agree and revised the Abstract accordingly. Rather than using simplified labels alone, we described the profiles in a way that better reflects their multidimensional health literacy patterns, including both areas of relative strength and areas of challenge.

6. Strengths and limitations of this study – did you plan to conduct causal inference and you couldn't do it?
   Response:
   We thank the Reviewer for this helpful point. No causal inference was planned in this study, given its cross-sectional design and descriptive, exploratory aims. In the revised "Strengths and limitations" section, we have clarified this point more explicitly and removed wording that might suggest that causal inference was intended but not achieved.

7. Introduction – the Ophelia process has 8 steps, not 9 steps. Phase 3 only has 2 steps.
   Response:
   We thank the Reviewer for noticing this error. We corrected the description of the Ophelia process accordingly and revise the relevant wording in the Introduction/Methods to reflect the correct 8-step structure.

8. Methods – p.44, line 53, 'foresees' may not be an appropriate word, can simply be 'uses'.
   Response:
   We agree and revised the wording accordingly.

9. Methods – the description about the 9 scales/dimensions of the HLQ is missing, as well as the score ranges.
Response:
We agree. In the revised Methods section, we added a concise but complete description of the nine HLQ dimensions and specify the score ranges for Part 1 (scales 1–5) and Part 2 (scales 6–9), so that readers can interpret the reported results without having to rely on external familiarity with the instrument.

10. Methods – while you have the details of the other questionnaires in the supplementary file, you still need to provide the score interpretations in your Methods section, for easier references.
Response:
We fully agree. In the revised manuscript, we reported the key score interpretation information for the additional instruments into the main Methods section, including the interpretive categories for NLit-IT, MEDI-LITE, and the relevant information for the Sense of Coherence scale, while keeping the supplementary material for more detailed instrument descriptions.

11. Results – 'financial status' is a better description than 'economic conditions'.
Response:
We agree and replaced "economic conditions" with "financial status" throughout the manuscript for greater clarity and consistency.

VERSION 2 - REVIEW

**Reviewer**	**1**
**Name**	**Hany, Mohamed**
**Affiliation**	**Alexandria University**
**Date**	**31-Mar-2026**
**COI**	

There are issues that remain unaddressed, partially addressed, or require further attention.

1. Changing the word "foresees" to "uses" (e.g., "Methods – p.44, line 53, 'foresees' may not be an appropriate word, can simply be 'uses'"). The authors responded, "We agree and revised the wording accordingly." However, the word "foresees" remains in the revised manuscript. From manuscript (Introduction): "The Ophelia process foresees three phases and eight steps [18, 19]: 1. identify strengths…"

2. The score ranges for the 9 scales/dimensions of the HLQ should be added to the Methods section. The authors stated, "In the revised Methods section, we added a concise but

complete description of the nine HLQ dimensions and specified the score ranges for Part 1 (scales 1–5) and Part 2 (scales 6–9)…" However, the score ranges (1-4 and 1-5) were not added to the main Methods section. They are mentioned only in the Results section and the Supplementary Table. From manuscript (Methods): "HLQ: it is a validated 44-item instrument covering nine dimensions of HL. It is a self-perceived measurement tool of HL with Likert-type items." (No score ranges are specified here).

3. The authors provide 95% confidence intervals for effect sizes ("Please provide effect sizes (e.g., η^2 for continuous variables, Cramer's V for categorical variables) and 95% CIs, not just p-values"). The authors agreed and stated that they added effect sizes. While effect sizes (Cramer's V, η^2, ω^2) were indeed added, the 95% Confidence Intervals (CIs) for these effect sizes are completely missing from the manuscript and tables.

4. sensitivity analyses comparing the primary imputation strategy with an alternative strategy and complete-case clustering. The authors responded by explaining their use of the Expectation-Maximization (EM) algorithm, but did not conduct or report the requested sensitivity analyses for imputation (e.g., complete-case vs. alternative imputation).

5. Expand instrument details for Sense of Coherence (SoC) and clarify if it was a mediator. The authors stated they expanded the description in the Methods section and clarified its role. However, crucial details like the Vogt et al. reference, the use of "nine bipolar adjective pairs", and the explicit statement that it was "not modelled as a mediator" were placed only in the Supplementary Material, not in the main manuscript body as implied.

6. If any cultural/practical adaptations were necessary for the MEDI-LITE tool. The authors stated they clarified this. However, the statement "No specific cultural adaptation of the instrument was required" was placed only in the Supplementary Material, not in the main text.

7. Typographical Error (Supplementary Table 1): In the description of the sociodemographic questionnaire within the supplementary material, there is a typographical error where "height" is misspelled as "eight". Supplementary Material: "…weight and eight)." (Should be "weight and height").

**Reviewer**	**2**
**Name**	**Cheng, Christina**
**Affiliation**	**La Trobe University**
**Date**	**16-Apr-2026**
**COI**	

Thank you for the revisions and I'm generally happy with your responses to my comments. I do notice that you sometimes have a 'strong' emphasis on the socioeconomic and behavioural gradients you found with the health literacy profiles, including employing

statistical analysis to support these claims. I wonder if it is the reason why the results kind of distract people from seeing that your aim was to identify health literacy profiles to develop fit-for-purpose interventions. The Conclusions of your Abstract started with a discussion about the gradients and even went onto propose potential 'intervention'. This should not be your Conclusions as the interventions were not yet co-designed. The Conclusions should focus on what you achieved with your study, echoing your aim. You Lay summary actually provides a clearer picture about what your study aim was. Also, the first paragraph of your Discussion is good in reminding readers that your aim was to produce health literacy profiles for intervention development.

With the 'Strengths and limitations of this study', I don't think you should include 'the cross-sectional design does not allow causal inferences' because you never set out to do any causal inferences. So you did even design a study that allowed you to do causal inferences. This is simply not applicable to your study.

I have a few more comments:

1. Methods – It is better to present the study instruments after the project set up and data collection, before discussion of statistical analysis. The 'Missing data handling', 'Clustering approach' and 'Statistical comparisons' should be part of 'Statistical analysis'.

2. Methods – The first paragraph under 'Study instruments' are not relevant to the instruments, they are more about description of your study.

3. Methods – The SoC is an instrument on its own. It's one of your study instruments. Shouldn't be mixed with your other demographic and health data. Did you use SOC-29 or SOC-13? Score range?

4. Methods – The score range and scoring of the HLQ should be presented in the section about the HLQ.

5. Methods – P. 6 Lines 9-14, those are more of your Results, not your Methods.

6. Methods – Clustering approach, please also include the distance metric used: squared Euclidean distance.

7. Methods – Clustering approach section, I don't think 'cluster sizes' is part of the consideration. The Ophelia cluster analysis may include small clusters, depending on the context. See p. 42 of the Ophelia manual the importance of including small clusters. Please note your other discussion about 'cluster sizes' in other parts of the manuscript.

8. Results – Table 2, please add in the score range of each variable in the table for easy interpretation of the results. Without an indication of score range, it's hard to interpret the results unless people are familiar with all these instruments.

9. Results – Include the scale number when describing the HLQ scales, e.g. Scale 3 'Actively managing my health' instead of just 'Actively managing my health'.

10. Results – Table 3, the heading for the HLQ scales in Table 3 should just be 'Scales', not 'Descriptive parameters'. You also don't need an extra column of 'HLQ scores by dimensions'. Do make sure you indicate the score range for the Part 1 and Part 2 scales.

11. Results – Table 4, also don't need the column 'HLQ scores by dimensions'. You also need to describe what the meaning of the colouring. See Box 10 of the Ophelia manual.

12. Results – You always report on 2 decimal points, but then switch to 3 decimal points sometimes, be consistent.

13. Results – with the presentation of your results of your clusters, you tend to also include what possible interventions are needed. Please note that these are interpretations of your results. They should be in your Discussion instead of Results which should just focus on the actual results. I see that you already have this discussion in the 'Discussion' section. Also, need to remind readers that these are your thinking, what interventions exactly needed will come from the people, from co-design.

14. Discussion – Second sentence of the second paragraph, I don't think this needs to be discussed when you clearly stated the scoring of the HLQ at the beginning.

15. Discussion – The paragraph (p. 16) starting with 'Implementation was supported by dedicated project staff…' This paragraph seems to jump out of the blue. Are you referring to 'implementation' of interventions, which follows the last paragraph discussion of intervention, or are you talking about implementation of this study? The first two sentences seem to be more about your current study. Don't seem to flow onto the next sentence about profile-informed actions. Please clarify.

16. Discussion – Last paragraph on p. 16 ' Implications for prevention are direct…' 'Prevention' of what? Not sure what you mean.

17. Discussion – Limitation about generalisability, while 'generalisability' may be limited, don't forget this study aimed to develop local interventions, so, your study purpose is not trying to find generalisable results. You planned to use these results to develop interventions for your specific context.

VERSION 2 - AUTHOR RESPONSE

Reviewer: 1
Dr. Mohamed Hany, Alexandria University
Comments to the Author:
There are issues that remain unaddressed, partially addressed, or require further attention.

1. **Changing the word "foresees" to "uses" (e.g., "Methods – p.44, line 53, 'foresees' may not be an appropriate word, can simply be 'uses'"). The authors responded, "We agree and revised the wording accordingly." However, the word "foresees" remains in the revised manuscript. From manuscript (Introduction): "The Ophelia process foresees three phases and eight steps [18, 19]: 1. identify strengths…"**

Thank you for pointing this out. We have now revised the wording and replaced "foresees" with "comprises" throughout the manuscript.

2. **The score ranges for the 9 scales/dimensions of the HLQ should be added to the Methods section. The authors stated, "In the revised Methods section, we added a concise but complete description of the nine HLQ dimensions and specified the score ranges for Part 1 (scales 1–5) and Part 2 (scales 6–9)…" However, the score ranges (1-4 and 1-5) were not added to the main Methods section. They are mentioned only in the Results section and the Supplementary Table. From manuscript (Methods): "HLQ: it is a validated 44-item instrument covering nine dimensions of HL. It is a self-perceived measurement tool of HL with Likert-type items." (No score ranges are specified here).**
   Thank you for pointing this out. We have now revised the Methods section to explicitly report the score ranges of the HLQ, specifying that scales 1–5 range from 1 to 4 and scales 6–9 from 1 to 5.

3. **The authors provide 95% confidence intervals for effect sizes ("Please provide effect sizes (e.g., η^2 for continuous variables, Cramer's V for categorical variables) and 95% CIs, not just p-values"). The authors agreed and stated that they added effect sizes. While effect sizes (Cramer's V, η^2, ω^2) were indeed added, the 95% Confidence Intervals (CIs) for these effect sizes are completely missing from the manuscript and tables.**
   Thank you for pointing this out. We have added 95% CIs for effect sizes.

4. **sensitivity analyses comparing the primary imputation strategy with an alternative strategy and complete-case clustering. The authors responded by explaining their use of the Expectation-Maximization (EM) algorithm, but did not conduct or report the requested sensitivity analyses for imputation (e.g., complete-case vs. alternative imputation).**
   We thank the Reviewer for this important clarification. We agree that, in the previous revision, we described the primary EM-based imputation strategy but did not adequately conduct and report the requested sensitivity analyses. We have now performed additional sensitivity analyses to assess whether the HLQ cluster solution was dependent on the missing-data handling approach.
   Specifically, we compared the primary EM-based strategy with: (i) an alternative within-scale mean imputation strategy, applying the same predefined HLQ missingness thresholds used in the primary analysis, and (ii) a complete-case clustering analysis restricted to participants with no missing HLQ item data. In both sensitivity analyses, hierarchical clustering was repeated using Ward's method on standardized HLQ scale scores, consistent with the primary clustering procedure.
   The alternative within-scale mean imputation analysis included 154 participants and produced a four-cluster solution with cluster sizes of 15, 50, 62, and 27 participants. The resulting profiles showed a clear and interpretable gradient across the nine HLQ domains, ranging from a lower-HL profile to intermediate profiles and a higher-HL profile. This pattern was broadly consistent with the primary EM-based solution.
   The complete-case analysis included 124 participants and also produced an interpretable four-cluster solution, with cluster sizes of 9, 40, 52, and 23 participants.

Compared with the primary EM-based analysis, the exact cluster sizes and composition differed, as expected given the exclusion of participants with partially missing HLQ data. However, the complete-case solution preserved the main substantive gradient across HLQ domains, distinguishing profiles with lower, intermediate, and higher HLQ scores.

Overall, these sensitivity analyses indicated that the main interpretation of heterogeneous HL profiles and a clear HLQ gradient was not driven solely by the EM imputation strategy. At the same time, we now acknowledge more explicitly that exact cluster membership and cluster sizes varied across missing-data strategies. We have added these analyses to the Methods and Results sections. We have also revised the Limitations to state that the four-cluster solution should be interpreted as exploratory and useful for local intervention planning within the Ophelia framework, rather than as a definitive latent classification.

5. **Expand instrument details for Sense of Coherence (SoC) and clarify if it was a mediator. The authors stated they expanded the description in the Methods section and clarified its role. However, crucial details like the Vogt et al. reference, the use of "nine bipolar adjective pairs", and the explicit statement that it was "not modelled as a mediator" were placed only in the Supplementary Material, not in the main manuscript body as implied.**
   Thank you for pointing this out. We have now revised the Methods section of the main manuscript to include the key details on the Sense of Coherence instrument. Specifically, we clarified that the scale used was an Italian adaptation based on nine bipolar adjective pairs originally developed by Vogt et al. We also explicitly clarified that SoC was not modelled as a mediator, but was used as a psychosocial correlate to support the interpretation of health literacy profiles.

6. **If any cultural/practical adaptations were necessary for the MEDI-LITE tool. The authors stated they clarified this. However, the statement "No specific cultural adaptation of the instrument was required" was placed only in the Supplementary Material, not in the main text.**
   Thank you for pointing this out. We have now revised the Methods section of the main manuscript to explicitly state that no specific cultural or practical adaptation of the MEDI-LITE instrument was required, as it has already been validated for use in the Italian population.

7. **Typographical Error (Supplementary Table 1): In the description of the sociodemographic questionnaire within the supplementary material, there is a typographical error where "height" is misspelled as "eight". Supplementary Material: "…weight and eight)." (Should be "weight and height").**
   Thank you for pointing this out. The typographical error in Supplementary Table 1 has now been corrected.

Reviewer: 2
Dr. Christina Cheng, La Trobe University
Comments to the Author:

Thank you for the revisions and I'm generally happy with your responses to my comments. I do notice that you sometimes have a 'strong' emphasis on the socioeconomic and behavioural gradients you found with the health literacy profiles, including employing statistical analysis to support these claims. I wonder if it is the reason why the results kind of distract people from seeing that your aim was to identify health literacy profiles to develop fit-for-purpose interventions. The Conclusions of your Abstract started with a discussion about the gradients and even went onto propose potential 'intervention'. This should not be your Conclusions as the interventions were not yet co-designed. The Conclusions should focus on what you achieved with your study, echoing your aim. You Lay summary actually provides a clearer picture about what your study aim was. Also, the first paragraph of your Discussion is good in reminding readers that your aim was to produce health literacy profiles for intervention development.
With the 'Strengths and limitations of this study', I don't think you should include 'the cross-sectional design does not allow causal inferences' because you never set out to do any causal inferences. So you did even design a study that allowed you to do causal inferences. This is simply not applicable to your study.

Thank you for this thoughtful comment. We agree that the primary aim of the study was to identify health literacy profiles to inform the development of fit-for-purpose interventions within the Ophelia framework, and that this should be clearly reflected in the framing of the manuscript. We have therefore revised the Conclusions of the Abstract to better align with the study aim and to avoid overemphasis on socioeconomic and behavioural gradients or premature interpretation in terms of interventions. We also refined the Discussion to maintain a clearer focus on the profiling objective.
Regarding the "Strengths and limitations" section, we acknowledge your point. The original statement referring to causal inference has been removed. Following comments from another reviewer, we included a revised wording indicating that the cross-sectional, descriptive design limits the temporal interpretation of the observed associations. We believe this formulation better reflects the descriptive nature of the study without implying that causal inference was an intended aim.

I have a few more comments:

1. **Methods – It is better to present the study instruments after the project set up and data collection, before discussion of statistical analysis. The 'Missing data handling', 'Clustering approach' and 'Statistical comparisons' should be part of 'Statistical analysis'.**
   Thank you for this suggestion. We have revised the Methods section accordingly, moving the description of the study instruments after the project set-up and data collection sections. We also reorganised the Methods to group "Missing data handling", "Clustering approach", and "Statistical comparisons" under a unified "Statistical analysis" subsection.

2. **Methods – The first paragraph under 'Study instruments' are not relevant to the instruments, they are more about description of your study.**
   Thank you for this comment. We agree that the first paragraph under "Study instruments" was not directly related to the description of the instruments. We have

therefore moved this paragraph to the "Project set-up and data collection" section to improve the structure and clarity of the Methods.

3. **Methods – The SoC is an instrument on its own. It's one of your study instruments. Shouldn't be mixed with your other demographic and health data. Did you use SOC-29 or SOC-13? Score range?**
   Thank you for this comment. We agree that the SoC should be presented as a separate study instrument. We have therefore revised the Methods section to report SoC independently from the sociodemographic and health-related questionnaire. We clarified that the instrument used is an Italian adaptation of a semantic differential scale based on nine bipolar adjective pairs. We also added a description of its scoring, specifying that each item is rated on a 7-point scale.

4. **Methods – The score range and scoring of the HLQ should be presented in the section about the HLQ.**
   Thank you for this comment. We have revised the HLQ section in the Methods to include both the score ranges and scoring procedure. Specifically, we now state that scales 1–5 range from 1 to 4, scales 6–9 range from 1 to 5, and that scale scores are calculated as the mean of the corresponding items, with higher scores indicating greater health literacy strengths in each dimension.

5. **Methods – P. 6 Lines 9-14, those are more of your Results, not your Methods.**
   Thank you for this comment. We agree that this information was more appropriately part of the Results section. We have therefore removed the description of the number of recruited and included participants from the Methods and moved it to the beginning of the Results section.

6. **Methods – Clustering approach, please also include the distance metric used: squared Euclidean distance.**
   Thank you for this comment. We have revised the Clustering approach section to explicitly specify the distance metric used, indicating that squared Euclidean distance was applied in the hierarchical cluster analysis.

7. **Methods – Clustering approach section, I don't think 'cluster sizes' is part of the consideration. The Ophelia cluster analysis may include small clusters, depending on the context. See p. 42 of the Ophelia manual the importance of including small clusters. Please note your other discussion about 'cluster sizes' in other parts of the manuscript.**
   Thank you for this important comment. We have revised the Clustering approach section to remove cluster size as a criterion for selecting the cluster solution, in line with the Ophelia framework. We clarified that the selection was based on agglomeration, within-cluster variability, and interpretability of the profiles. We also reviewed the manuscript to ensure that cluster size is not used as a decision criterion, acknowledging that smaller clusters may represent important vulnerable subgroups.

8. **Results – Table 2, please add in the score range of each variable in the table for easy interpretation of the results. Without an indication of score range, it's hard to interpret the results unless people are familiar with all these instruments.**
   Thank you for this comment. We have revised Table 2 to include the score range for each variable, in order to facilitate interpretation of the results.

9. **Results – Include the scale number when describing the HLQ scales, e.g. Scale 3 'Actively managing my health' instead of just 'Actively managing my health'.**
   Thank you for this suggestion. We have revised the Results section to include the HLQ scale number whenever individual HLQ scales are described, to improve clarity and consistency.

10. **Results – Table 3, the heading for the HLQ scales in Table 3 should just be 'Scales', not 'Descriptive parameters'. You also don't need an extra column of 'HLQ scores by dimensions'. Do make sure you indicate the score range for the Part 1 and Part 2 scales.**
    Thank you for this comment. We have revised Table 3 by simplifying the headings, removing the redundant "HLQ scores by dimensions" column, labelling the first column as "Scales", and adding the score range for each HLQ scale to clarify the distinction between Part 1 and Part 2 scales.

11. **Results – Table 4, also don't need the column 'HLQ scores by dimensions'. You also need to describe what the meaning of the colouring. See Box 10 of the Ophelia manual.**
    Thank you for this comment. We have revised Table 4 by removing the redundant "HLQ scores by dimensions" column and by adding an explanatory note describing the meaning of the colour shading, in line with the Ophelia manual.

12. **Results – You always report on 2 decimal points, but then switch to 3 decimal points sometimes, be consistent.**
    Thank you for this comment. We have revised the manuscript to ensure consistent reporting of numerical values, standardising all results to two decimal places throughout the text and tables.

13. **Results – with the presentation of your results of your clusters, you tend to also include what possible interventions are needed. Please note that these are interpretations of your results. They should be in your Discussion instead of Results which should just focus on the actual results. I see that you already have this discussion in the 'Discussion' section. Also, need to remind readers that these are your thinking, what interventions exactly needed will come from the people, from co-design.**
    Thank you for this important comment. We agree that the Results section should focus on the presentation of findings only. We have therefore revised the Results to remove interpretative statements and references to potential interventions, retaining a purely descriptive presentation of the cluster profiles.

14. **Discussion – Second sentence of the second paragraph, I don't think this needs to be discussed when you clearly stated the scoring of the HLQ at the beginning.**
    Thank you for this comment. We agree that this explanation is no longer needed in the Discussion now that the HLQ scoring and score ranges have been clearly described in the Methods section. We have therefore removed this sentence from the Discussion.

15. **Discussion – The paragraph (p. 16) starting with 'Implementation was supported by dedicated project staff…' This paragraph seems to jump out of the blue. Are you referring to 'implementation' of interventions, which follows the last paragraph discussion of intervention, or are you talking about implementation of this study? The first two sentences seem to be more about your current study. Don't seem to flow onto the next sentence about profile-informed actions. Please clarify.**
    Thank you for this comment. We have revised the paragraph to clarify that "implementation" refers to the implementation of profile-informed interventions, as suggested by the reviewer. We also removed wording that could be interpreted as referring to the implementation of the present study and improved the flow from the preceding discussion on intervention development.

16. **Discussion – Last paragraph on p. 16 ' Implications for prevention are direct…' 'Prevention' of what? Not sure what you mean.**
    Thank you for this comment. We have revised the sentence to clarify that we are referring specifically to cardiovascular prevention planning, rather than prevention in general.

17. **Discussion – Limitation about generalisability, while 'generalisability' may be limited, don't forget this study aimed to develop local interventions, so, your study purpose is not trying to find generalisable results. You planned to use these results to develop interventions for your specific context.**
    Thank you for this comment. We agree that the primary aim of the study was to generate locally meaningful health literacy profiles to inform the development of fit-for-purpose interventions within the Ophelia framework, rather than to produce generalisable estimates. However, we retained the explicit discussion on generalisability in response to Reviewer 1's request to clearly distinguish between context-specific and potentially transferable findings. We believe that the current wording reflects both perspectives, acknowledging the local focus of the study while also providing transparency regarding the potential transferability of the approach.

VERSION 3 - REVIEW

**Reviewer**	**1**
**Name**	**Hany, Mohamed**
**Affiliation**	**Alexandria University**
**Date**	**18-May-2026**

COI

Minor issues:

- Table 3 still does not indicate the theoretical score range for each HLQ scale or clearly distinguish the Part 1 and Part 2 scale ranges within the table. The table heading now uses "Scales," but the columns are only "Mean," "Standard Deviation," "Minimum," "Maximum," and percentiles; these are observed values, not the requested score ranges. The manuscript only states before the table: "response ranges of the HLQ (1-4 for the scales 1-5, 1-5 for the scales 6-9)", but the table itself does not include this score-range information.

- The Results section still does not consistently include the HLQ scale number whenever an individual HLQ scale is described. For example, one sentence identifies the lowest score as Scale 7, but gives the highest score without the scale number: "while the highest for 'Understand health information well enough to know what to do' (3.65 ±0.67)." This should identify it as Scale 9 for consistency.

- Numerical formatting remains inconsistent despite the response stating that all results were standardized to two decimal places. The clean manuscript still includes one-decimal reporting, such as "6.6 (1.0)" and "44.7", and three-decimal reporting, such as "0.342," "0.409," "0.351," "0.201 to 0.258," and "ω^2 = 0.178."

**Reviewer**	**2**
**Name**	**Cheng, Christina**
**Affiliation**	**La Trobe University**
**Date**	**23-May-2026**
**COI**	

Thank you for revising the manuscript again. This new revision is much more coherent and well-presented. The Introduction provides a solid foundation for your study and the Discussion is thorough. I only have a few more minor comments:

1. The subheading of 2.1 indicates recruitment period will be presented, but recruitment period was reported in Section 2.2.

2. Section 2.3, first sentence, written informed consent has already been mentioned at the start of the Methods section.

3. Section 2.3, the dot point relates to the HLQ should start with 'Health Literacy Questionnaire (HLQ):'

4. Section 2.4.3, the sentence 'All the analyses were conducted using IBM SPSS 28.0.' should apply to your other analyses, is that correct? If yes, it should be at the start of Section 2.4 Statistical analysis.

5. Please note that there are still inconsistencies in using 2 decimal points or 3 decimal points.

6. I'd suggest including the score ranges for Tables 3 and 4 for easy score interpretation.

7. In the description of Cluster 4, this phrase 'should be interpreted as indicating that these tasks were only sometimes easy and still often difficult, rather than reflecting a clearly strong ability.' can be confusing. You have already indicated that the score range is 1-5, therefore, people would not interpret it as 'strong ability'. By saying 'rather than reflecting a clearly strong ability' will only confuse your readers.

VERSION 3 - AUTHOR RESPONSE

Reviewer: 1
Dr. Mohamed Hany, Alexandria University
Comments to the Author:
Minor issues:
- Table 3 still does not indicate the theoretical score range for each HLQ scale or clearly distinguish the Part 1 and Part 2 scale ranges within the table. The table heading now uses "Scales," but the columns are only "Mean," "Standard Deviation," "Minimum," "Maximum," and percentiles; these are observed values, not the requested score ranges. The manuscript only states before the table: "response ranges of the HLQ (1-4 for the scales 1-5, 1-5 for the scales 6-9)", but the table itself does not include this score-range information.
Thank you for this helpful clarification. We agree that the previous revision did not fully address the request. We have now revised Table 3 by adding a dedicated column entitled "Theoretical score range" and by indicating the relevant range for each HLQ scale. Specifically, scales 1–5 are now shown as having a theoretical score range of 1–4, while scales 6–9 are shown as having a theoretical score range of 1–5.

- The Results section still does not consistently include the HLQ scale number whenever an individual HLQ scale is described. For example, one sentence identifies the lowest score as Scale 7, but gives the highest score without the scale number: "while the highest for 'Understand health information well enough to know what to do' (3.65 ±0.67)." This should identify it as Scale 9 for consistency.
Thank you for this helpful comment. We agree that HLQ scale numbers should be reported consistently whenever individual HLQ scales are described. We have therefore carefully revised the Results section and added the corresponding HLQ scale number wherever a specific HLQ scale or domain is mentioned.

- Numerical formatting remains inconsistent despite the response stating that all results were standardized to two decimal places. The clean manuscript still includes one-decimal reporting, such as "6.6 (1.0)" and "44.7", and three-decimal reporting, such as "0.342," "0.409," "0.351," "0.201 to 0.258," and "ω^2 = 0.178."
Thank you for pointing this out. We agree that numerical formatting was still inconsistent in the clean version of the manuscript. We have now carefully revised the manuscript,

including the Results section and all relevant tables, to ensure consistent numerical reporting.

Reviewer: 2
Dr. Christina Cheng, La Trobe University
Comments to the Author:
Thank you for revising the manuscript again. This new revision is much more coherent and well-presented. The Introduction provides a solid foundation for your study and the Discussion is thorough. I only have a few more minor comments:
1. The subheading of 2.1 indicates recruitment period will be presented, but recruitment period was reported in Section 2.2.
Thank you for noting this inconsistency. We have revised the subheading of Section 2.1 to better reflect its content, since the recruitment period is reported in Section 2.2.

2. Section 2.3, first sentence, written informed consent has already been mentioned at the start of the Methods section.
Thank you for pointing this out. We agree that the reference to written informed consent in Section 2.3 was redundant, as this information had already been provided at the beginning of the Methods section. We have therefore removed the repeated mention from Section 2.3.

3. Section 2.3, the dot point relates to the HLQ should start with 'Health Literacy Questionnaire (HLQ):'
Thank you for this suggestion. We have revised the bullet point in Section 2.3 so that the HLQ instrument is introduced explicitly and consistently with the other study instruments.

4. Section 2.4.3, the sentence 'All the analyses were conducted using IBM SPSS 28.0.' should apply to your other analyses, is that correct? If yes, it should be at the start of Section 2.4 Statistical analysis.
Thank you for this suggestion. We confirm that IBM SPSS Statistics version 28.0 was used for all analyses described in Section 2.4. We have therefore moved this sentence from Section 2.4.3 to the beginning of Section 2.4, so that it clearly applies to the entire statistical analysis section.

5. Please note that there are still inconsistencies in using 2 decimal points or 3 decimal points.
Thank you for noting this remaining inconsistency. We have carefully rechecked the manuscript and corrected the numerical formatting throughout the text and tables.

6. I'd suggest including the score ranges for Tables 3 and 4 for easy score interpretation.
Thank you for this useful suggestion. We have revised both Table 3 and Table 4 to make the theoretical HLQ score ranges clearer. In Table 3, we added a dedicated column reporting the theoretical score range for each scale. In Table 4, we included the theoretical score range directly below each HLQ scale label.

7. In the description of Cluster 4, this phrase 'should be interpreted as indicating that these tasks were only sometimes easy and still often difficult, rather than reflecting a clearly

strong ability.' can be confusing. You have already indicated that the score range is 1-5, therefore, people would not interpret it as 'strong ability'. By saying 'rather than reflecting a clearly strong ability' will only confuse your readers.

Thank you for this clarification. We agree that the phrase could be confusing and was unnecessary given that the score range is now clearly indicated. We have therefore simplified the sentence and removed the wording "rather than reflecting a clearly strong ability."
